# Reconstruction of the Evolutionary Histories of UGT Gene Superfamily in Legumes Clarifies the Functional Divergence of Duplicates in Specialized Metabolism

**DOI:** 10.3390/ijms21051855

**Published:** 2020-03-08

**Authors:** Panneerselvam Krishnamurthy, Chigen Tsukamoto, Masao Ishimoto

**Affiliations:** 1 Institute of Crop Science, NARO, 2-1-2 Kannondai, Tsukuba 305-8518, Japan; 2 Faculty of Agriculture, Iwate University, Morioka 020-8550, Japan

**Keywords:** family 1 glycosyltransferases, legumes, putative ortholog loci, soyasaponins, specialized metabolites, triterpenoids

## Abstract

Plant uridine 5′-diphosphate glycosyltransferases (UGTs) influence the physiochemical properties of several classes of specialized metabolites including triterpenoids via glycosylation. To uncover the evolutionary past of UGTs of soyasaponins (a group of beneficial triterpene glycosides widespread among Leguminosae), the UGT gene superfamily in *Medicago truncatula*, *Glycine max*, *Phaseolus vulgaris*, *Lotus japonicus,* and *Trifolium pratense* genomes were systematically mined. A total of 834 nonredundant UGTs were identified and categorized into 98 putative orthologous loci (POLs) using tree-based and graph-based methods. Major key findings in this study were of, (i) 17 POLs represent potential catalysts for triterpene glycosylation in legumes, (ii) UGTs responsible for the addition of second (*UGT73P2*: galactosyltransferase and *UGT73P10*: arabinosyltransferase) and third (*UGT91H4*: rhamnosyltransferase and *UGT91H9*: glucosyltransferase) sugars of the C-3 sugar chain of soyasaponins were resulted from duplication events occurred before and after the hologalegina–millettoid split, respectively, and followed neofunctionalization in species-/ lineage-specific manner, and (iii) UGTs responsible for the C-22-*O* glycosylation of group A (arabinosyltransferase) and DDMP saponins (DDMPtransferase) and the second sugar of C-22 sugar chain of group A saponins (*UGT73F2*: glucosyltransferase) may all share a common ancestor. Our findings showed a way to trace the evolutionary history of UGTs involved in specialized metabolism.

## 1. Introduction

Glycosyltransferases (GTs) (EC 2.4.x.y) are ubiquitous enzymes of a superfamily that generally mediate the transfer of carbohydrate moieties from nucleotide-activated donor molecules to a broad range of saccharide or non-saccharide acceptor molecules and form glycosidic linkages via two distinct catalytic mechanisms-defined inversion or retention [1,2]. They are present in all phyla and influence the physio-chemical properties of acceptor molecules through which entail in diverse pivotal cellular processes [3]. Though GTs are extremely divergent in terms of sequence similarity, most of its members exhibit well-conserved secondary and tertiary structures and adopt either the characterized GT-A or GT-B fold [1,4,5,6].

The carbohydrate-active enzyme (CAZy) database classifies the GTs from diverse species based on their amino acid sequence conservation [7]. As of March 2020, a total of 110 numbered GT families have been identified and the number will likely increase in the future (http://www.cazy.org/GlycosylTransferases.html). Of these, GTs utilizing uridine 5′-diphosphate (UDP)-conjugated carbohydrates as the sugar donors are referred as family 1 GTs (alias UGTs). They are generally cytosolic in nature, widespread in the plant kingdom, and constitute the largest GT family [8,9]. Plant UGTs are assigned between families 71–100, 701–1000 and 7001–10000 in the current classification system (https://prime.vetmed.wsu.edu/resources/udp-glucuronsyltransferase-homepage). They are inverting GTs exhibiting GT-B fold and consist of a characteristic 44-amino acid consensus sequence, designated as the plant secondary product glycosyltransferase (PSPG) box, at the C terminus [10,11]. The highly divergent N-terminal and the well-conserved C-terminal PSPG box are acknowledged to be engaged in the determination of sugar acceptor and sugar donor, respectively [11]. Plant UGTs glycosylate multitude of acceptor molecules including phytohormones and diverse specialized metabolites by which influence the acceptor molecules stability, solubility, storage, transport, compartmentalization, and bioactivity [8,10,12,13]. They also have important functions in detoxification of xenobiotics and facilitate plant protection [8,14,15].

Plants naturally synthesize a tremendous number of triterpenoids through specialized metabolism that often exists as glycosidic conjugates (i.e., saponins) and have potential functions in different sectors of day-to-day life applications [16,17,18]. Like that of steroids, the committed biosynthesis pathway of triterpenoids stems from the mevalonate pathway-derived precursor 2,3-oxidosqualene [19]. Several triterpene scaffolds generate from 2,3-oxidosqualene by one of many oxidosqualene cyclase (OSC) enzymes, but the OSC namely β-amyrin synthase yields the most common scaffold β-amyrin [20,21]. The members of cytochrome P450 monooxygenase (CYP450) and UGT families decorate the pentacyclic C_30_ skeleton of β-amyrin by oxygenation and glycosylation, respectively, at various active sites depending on the genetic background of the given genera/species. Though the vast diversity of triterpenoids is broadly achieved by OSCs, CYP450s, and UGTs, the diversification created by UGTs is exponential and by far the most. For example, in soybean (*Glycine max*), the combinatorial activity of three different CYP450s produces only two soyasapogenols (namely A and B) from β-amyrin whereas the epistatic activity of eight different UGTs on soyasapogenol A (SA) and B (SB) could generate >50 triterpene glycosides [22]. Triterpene-related UGTs not only enhance the diversification of triterpenoids and its pharmacological values, but are also involved in plant defense against take-all-diseases [23] and herbivores [24].

Soyasaponins are oleanane-type pentacyclic triterpene glycosides implicated in diverse pharmaceutical benefits [25], several characters of root growth [26] and in undesirable taste properties of soybean-based food products [27]. They are widespread among the species of Leguminosae including the model legumes barrel medic (*Medicago truncatula*) and birdsfoot trefoil (*Lotus japonicus*), but abundant principally in the seeds of *G. max*. At least nine different UGTs have been assumed to be involved in the biosynthesis of soyasaponins. Of these, seven UGTs have been characterized to date (*UGT73P2* and *UGT91H4* [28], *UGT73F2* and its allelic variant *UGT73F4* [29], *UGT73P10* [30], *UGT91H9* [31], and *UGT73B4* [32]), excluding the UGTs responsible for the C-3-*O*- and C-22-*O*-glycosylation of SA/SB and SA respectively. Though the biochemical and genetical characteristics of soyasaponin UGTs are studied well, how they evolved upon large-scale [whole-genome duplication (WGD) alias polyploidization)] or small-scale (e.g., segmental/ tandem) duplication events remains to be studied. Also, the corresponding homologs of soyasaponin UGTs in the model legumes *M. truncatula*/*L. japonicus* are yet to be discovered.

Leguminosae (alias Fabaceae) is the third largest flowering plant family consists of more than 750 genera and 19,500 species [33]. Leguminosae plants biosynthesize a vast diversity of specialized metabolites as glycosidic conjugates in taxa-specific manner [34]. Both the model legumes *M. truncatula* and *L. japonicus*, as well as the economically important oil seed legume crop *G. max,* all belong to a legume subfamily Papilionoidea which experienced two WGD events—one at ~59 [papilionoid-specific WGD (PWGD)] and the other at ~13 [glycine-specific WGD (GWGD)] million years ago (MYA) [35]. To explore the effect of WGD events on soyasaponin UGTs, a systematic genome-wide survey of UGT gene superfamily was conducted using the latest genome versions of *M. truncatula* (MtUGTs), *G. max* (GmUGTs), *L. japonicus* (LjUGTs), common bean (*Phaseolus vulgaris*; PvUGTs), and red clover (*Trifolium pratense*; TpUGTs). All the identified UGTs were assigned to putative ortholog loci (POLs) for the first time, which disclosed the mode of expansion of UGTs, gene gain/loss and intron addition/deletion events in *M. truncatula* and *G. max*. POL assignments underscore the evolutionary origin of soyasaponin UGTs and functional divergence of their homologs. In addition, it showed a way for future studies to easily pick up candidate ortholog UGTs across legumes to unravel their functions and extends our understanding in the evolution of UGT gene family.

## 2. Results

### 2.1. Genome-Wide Identification of UGT Gene Family in Five Papilionoid Legumes

With the help of PSPG sequence and several other criteria (see Materials and Methods Section 4.1), a total of 243, 208, 168, 94, and 121 authentic UGTs were identified for *M. truncatula*, *G. max*, *P. vulgaris*, *L. japonicus,* and *T. pratense* respectively in this study (Table 1). These numbers shall be treated as the least because many sequences (36 for *M. truncatula*, 34 for *G. max*, 5 for *P. vulgaris*, 64 for *L. japonicus* and 50 for *T. pratense*) in all five species were excluded based on one or more criteria (Tables S1–S5). Following the guidelines of UGT nomenclature committee, no UGTs were named in this study because we believe that the final designation of their nomenclature should be made after their functional characterization by in vitro and/or in vivo techniques.

The UGT family of *G. max* [36,41,46] and *L. japonicus* [47] has been described previously. We did not go for a detailed comparison with the results of Yin et al. [46,47] because of discrepancies in the screening criteria of those studies (e.g., they considered all proteins having PSPG motif as UGTs irrespective of the protein length) with that of the current study. Though Caputi et al. [36] and Rehman et al. [41] utilized the first genome version of *G. max*, the former identified 183 UGTs while the latter identified 149 UGTs. Since it remains unclear how Rehman et al. [41] underestimated the number of GmUGTs, we compared our results with that of Caputi et al. [36]. Out of 183, 160 sequences were also identified in this study; seven sequences were redundant, 16 were absent, and 48 were new in the second *G. max* genome assembly (Wm82.a2.v1). This suggests that the number of UGTs identified in this study may vary in the future genome assemblies of the corresponding species.

### 2.2. Phylogenetic Relationship of the UGTs in Five Papilionoid Legumes

**Figure 1:**
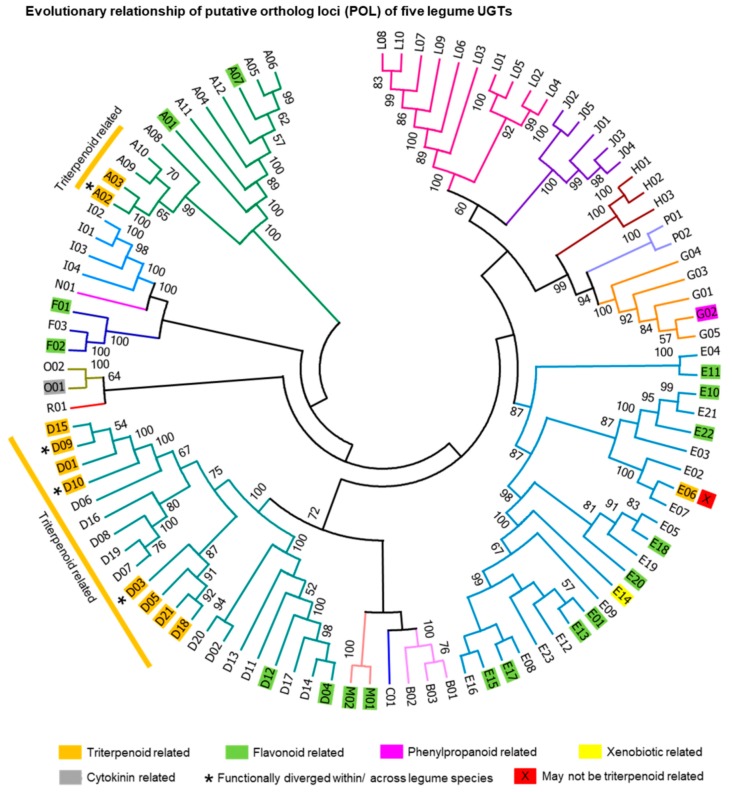
Evolutionary relationship of putative ortholog loci (POL) of five legume UGTs. A total of 196 full-length amino acid sequences covering all phylogenetic groups (A–R, excluding K and Q) and POLs (*n* = 98) were selected from *Glycine max* (number of UGTs = 86), *Medicago truncatula* (76), *Phaseolus vulgaris* (9), *Lotus japonicus* (8), *Cajanus cajan* (3), *Trifolium pratense* (3), *Vigna radiata* (3), *Cicer arietinum* (2), *Arachis duranensis* (4), *Lupinus angustifolius* (1), and *Trifolium subterraneum* (1). Each POL included two sequences, each from different species (See Text S1). Subtrees (i.e., UGT pairs) were compressed with corresponding POL numbers to understand the POL relationship. POLs highlighted in orange, green, purple, yellow, and gray backgrounds denote that at least one UGT from that POL has been characterized for the glycosylation of triterpenoids, flavonoids, phenylpropanoids, xenobiotics, and cytokinin’s respectively. The first letter of each POL represents their phylogenetic groups.

Plant UGTs from diverse species could form at least 18 distinct groups (designated A–R) in unrooted phylogenetic analyses (Table 1). Earlier studies identified 14 (A–N) of the 18 UGT groups using Arabidopsis genome [36]. Perhaps, whole-genome examination from other higher plants identified four new UGT groups named O–R: groups O and P observed in many higher plants including rice [36] while the existence of groups Q (only in maize [45] and wheat [44]) and R (only in tea [37]) are restricted. In this study, the five-legume species found to retain 14–16 phylogenetic groups (A–R, except K and Q) (Figure 1; Table 1). Notably, (i) all the five legumes lacked groups K and Q, (ii) group C members only found in *P. vulgaris*, and (iii) groups N and R absent respectively in *T. pratense* and *G. max*. Interestingly, search in other legumes identified group K members only in Arachis species, group C members in pigeon pea (*Cajanus cajan*) and Vigna species while no legume species carried group Q. This suggests many legumes lost groups K and C during their course of evolution, and the presence of group Q could be specific to monocots. The number of individuals within each group among the five legumes has varied (Table 1). Nevertheless, the highest number of UGTs was observed in groups E and D followed by groups L, G, and A. This coincides with Caputi et al. [36] that those five groups in each species have expanded more than any other groups during the evolution of higher plants. Among the five legumes, *M. truncatula* had relatively many members in groups G and L while *G. max* and *P. vulgaris* had that in group I, suggesting that those groups may have expanded evolutionarily in species- and lineage-specific manner, respectively.

### 2.3. Putative Ortholog Loci Assignments for UGTs of Papilionoid Legumes

Although *M. truncatula*, *P. vulgaris*, *L. japonicus,* and *T. pratense* have undergone similar WGD events, the retention of a high number of UGTs in *M. truncatula* (Table 1) suggest that MtUGT family may have expanded through multiple species-specific small-scale duplication events during its evolution course. Concurrently, despite the recent GWGD event, *G. max* retained relatively less UGTs than *M. truncatula*. This implies *G. max* may have lost several UGTs during its evolution. To validate these presumptions and to trace the evolutionary histories of UGTs in legumes, assigning putative ortholog loci (POL) is essential. Because legumes experienced different duplication events, the gene number may vary among the species, but the gene loci number would be evolutionarily more stable. Several platforms such as POG [48] and PLAZA [49] were developed to trace the cluster of ortholog groups among species using different criteria including the genome/gene synteny search between species. Additionally, the Phytozome gene family [50] and context viewer in Legume Information System (LIS) database [51] were helpful to get basic insight into orthologs but not feasible when the gene family has too many duplicates. These platforms, databases, and tools were certainly helpful but not sufficient to confidently assign POL for all the identified legume UGTs due to several species-specific duplication events. After several trial and error attempts to subdue the shortcomings in POL assignments, we observed that the multi-species phylogenetic clustering and the full-length amino acid percent identity were together effective in assigning POL for legume UGTs.

Using the proposed scenario (see Materials and Methods Section 4.3), 98 POLs were estimated by combining *M. truncatula*, *G. max*, *P. vulgaris*, *L. japonicus,* and *T. pratense* UGTs (Table S6; Figures S1–S11). Of these, 35 POLs had members in all five species, 25 POLs had members in either of four species, 16 POLs had members in either of three species, and 12 POLs had members in either of two species (Figure S12). Albeit using five different yet closely related species, ten POLs (one each for *M. truncatula* and *L. japonicus*, two for *T. pratense*, and three each for *G. max* and *P. vulgaris*) lacked corresponding orthologs within the five species (Figure S12). They were assigned to POL based on the UGTs of other legume species such as pigeon pea and mung bean (*Vigna radiata*) (Table S6). This suggests that analyzing UGT family of other papilionoid and non-papilionoid species may reveal new POLs. Furthermore, members in some POLs (e.g., E20 and L04) shared relatively less amino acid identity with their co-members [they were included in the same POL due to the absence of true orthologs in other legumes (Table S6)], and members in some POLs (e.g., D03 and G02) formed large clusters with complex relationship while in some POLs (e.g., D02, D06 and I03) they formed short clusters. These imply that some of the current POLs can be divided into more POLs or combined into other existing POLs in future and therefore the number of POLs identified in this study should be treated as the least.

Of the 98 POLs, the highest number of POL was found for the major groups E (*n* = 23) and D (*n* = 21), as the number of UGTs present in these groups was high. Groups A and L sustained respectively 12 and 10 POLs while all the remaining groups sustained 1–5 POLs (Table 2 and Table S6). Noteworthy is that albeit the number of UGTs in groups G and J had huge difference, both groups consisted of only 5 POLs each. Further observation clearly showed that the retention, expansion, or lose of POL in each phylogenetic group was merely species-specific followed by lineage-specific. For example, (i) groups G and L in *M. truncatula* had only 5 and 9 POLs but contained 39 and 33 UGTs, respectively, reflecting the species-specific expansion; because, such expansion was not observed for *G. max*, *P. vulgaris*, *L. japonicus* and *T. pratense*; and (ii) group I in *G. max* and *P. vulgaris* retained 15–17 UGTs in 4 POLs whereas *M. truncatula*, *L. japonicus* and *T. pratense* retained 1–2 POLs with 1–5 UGTs suggesting that the expansion of group I was specific to *G. max*/*P. vulgaris* lineage.

### 2.4. Expansion of UGTs in M. truncatula and G. max

Because of the sequencing coverage, completeness, and higher resolution, we only focused MtUGTs and GmUGTs from here for all further analyses with fewer exceptions. POL assignments revealed an interesting criterion: the 243 UGTs of *M. truncatula* traced back to 76 POLs whereas that of 208 GmUGTs were traced back to 86 POLs (Table 2; Table S6). This emphasizes the fact that UGT family in *M. truncatula* expanded more but lost some POLs during its evolution. Perhaps, our findings show that the 76 POLs in *M. truncatula* were dispersed as 33 single-copy, 14 double-copy, and 29 multi-copy POLs (Table S6). In *G. max*, 40 were single-copy, 21 were double-copy, and 25 were multi-copy POLs. Notably, 19 POLs were single-copy in both species. In *M. truncatula*, four multi-copy POLs namely G02, D03, D06, and L01 had 30, 17, 15, and 11 members respectively (Table S6). These four POLs represent 30% of UGTs in total number of MtUGTs (73 in 243) whereas that represent only 8.7% in *G. max* (18 in 208). We thus attributed these four POLs as the predominant source for the higher number of UGTs in *M. truncatula*. No UGT members had been found for 22 POLs in *M. truncatula* and 12 POLs in *G. max*. Of these, 16 POLs had no members in *M. truncatula* but had in *G. max* whereas 6 POLs had no members in *G. max* but had in *M. truncatula*; 6 POLs lacked members from both species. This shows that the retention or loss of POLs in *M. truncatula* and *G. max* was species- or lineage-specific.

### 2.5. Analysis of Intron Gain/Loss Events in M. truncatula and G. max

Introns present in the coding sequences were considered for this study. The majority of UGTs in *M. truncatula* (*n* = 140; 57.6%) and *G. max* (114; 54.8%) had no introns. Among the intron containing UGTs, 87 out of 103 (84.5%) in *M. truncatula* and 76 out of 94 in *G. max* (80.9%) had one intron. Nine UGTs contained 2, five contained 3, and two contained 5 introns in *M. truncatula* (Table S1) while *G. max* had two introns in 11, three in 5, and four in 2 UGTs (Table S2).

Intron gain or loss events were inferred by the comparison of members present in the given POL across five legumes (Table S7). The 98 POLs of UGTs were first classified into three types: no-intron POLs (*n* = 42), one-intron POLs (*n* = 26) and mixed-intron POLs (*n* = 30). Based on our criteria (see Materials and Methods Section 4.4), 11 one-intron UGTs from *M. truncatula,* and 9 one-intron UGTs from *G. max* were found as intron-gained genes. This implies that 12.6% (11 in 87) of one-intron MtUGTs and 11.8% (9 in 76) of one-intron GmUGTs gained introns evolutionarily. Though experimental validation is required, this finding suggest that no-intron UGTs can become one-intron UGTs evolutionarily. In addition, 16 MtUGTs and 18 GmUGTs were also identified as intron-gained genes which consisted of 2–5 introns. Our findings reveal that, six (G, H, I, J, N, and P) and two (O and R) phylogenetic groups could be designated as one-intron and no-intron containing groups, respectively.

### 2.6. Chromosomal Locations and Gene Duplication Analyses in M. truncatula and G. max

UGTs distributed throughout all the chromosomes (Ch) of *M. truncatula* (Figure S13; Table S1) and *G. max* (Figure S14; Table S2). The UGTs density per chromosome was highly uneven in both species. In *M. truncatula*, Ch6 (*n* = 42) had the highest number of UGTs followed by Ch5/Ch8 (*n* = 40) and Ch7 (*n* = 37). Ch1 (*n* = 14) and Ch3 (*n* = 15) had the least number of UGTs, while Ch2 and Ch4 had 20 and 25 UGTs, respectively. In *G. max*, Ch8 had the maximum number of UGTs (*n* = 21) followed by Ch3 (*n* = 18) and Ch2 (*n* = 17), whereas the least number of UGTs found in Ch4, Ch5, Ch17, and Ch20 which had 3–4 UGTs. All other chromosomes had 6–16 UGTs. Scaffolds represent 10 and one UGTs in *M. truncatula* and *G. max* respectively.

All the double-copy and multi-copy POLs in *M. truncatula* and *G. max* were selected for gene duplication analysis (Table S8). The members in double-copy POLs shared 60–94% amino acid identity at full-length protein level whereas that of multi-copy POLs shared 54–99% within each POL in both species. The variation in sequence conservation among UGTs in the given POL suggest that the duplicated copies diverged rapidly after the duplication. In *M. truncatula*, 61 sequences were identified as segmental duplicates and 149 sequences were identified as tandem duplicates. This shows that the UGT family in *M. truncatula* has been expanded majorly through tandem duplication (61.3%; 149 in 243) and subtly through segmental duplication (25.1%; 61 in 243) events. A similar trend was observed for the UGT family expansion in *G. max*, in which tandem duplication contributed 51.4% (107 in 208) whereas segmental duplication contributed 29.3% (61 in 208). Of the 29 multi-copy POLs in *M. truncatula*, eight POLs (A06, A10, E10, I02, J02, L07, L08, and P01) involved only in tandem duplication events; four POLs (A08, D02, G05, and L04) involved only in segmental duplication events; and the remaining 17 POLs experienced both events (Table S8). Among the 25 multi-copy POLs in *G. max*, nine POLs (A01, D03, D04, D06, D07, E10, E13, E19, and I02) involved only in tandem duplication, one POL (E17) involved only in segmental duplication and the remaining 15 POLs involved in both events. By using chromosomal positions and the gene order, it appears that the members in 17 multi-copy POLs (which experienced both events) in *M. truncatula* were first scattered on different *M. truncatula* chromosomes via segmental duplication and then concentrated through tandem duplication (e.g., D06). Whereas, it appears that most members (if not all) of 15 multi-copy POLs (which experienced both events) in *G. max* were first underwent tandem duplication and then translocated into other chromosomes by segmental duplication or by GWGD (e.g., D03).

### 2.7. Duplication History and Functional Divergence of Triterpene Related UGT POLs in M. truncatula and G. max

Albeit the genomes of *M. truncatula* and *G. max* retained hundreds of putative UGT sequences, only a handful of them have been studied for their functions (10 in *M. truncatula* and 27 in *G. max*) to date. In the case of triterpene glycosylation, only three MtUGTs and eight GmUGTs were characterized. These 11 UGTs were clustered and evolutionarily close to 11 POLs (A02, A03, D01, D03, D05, D09, D10, D15, D18, D21, and E06) (Figure 1).

**Figure 2:**
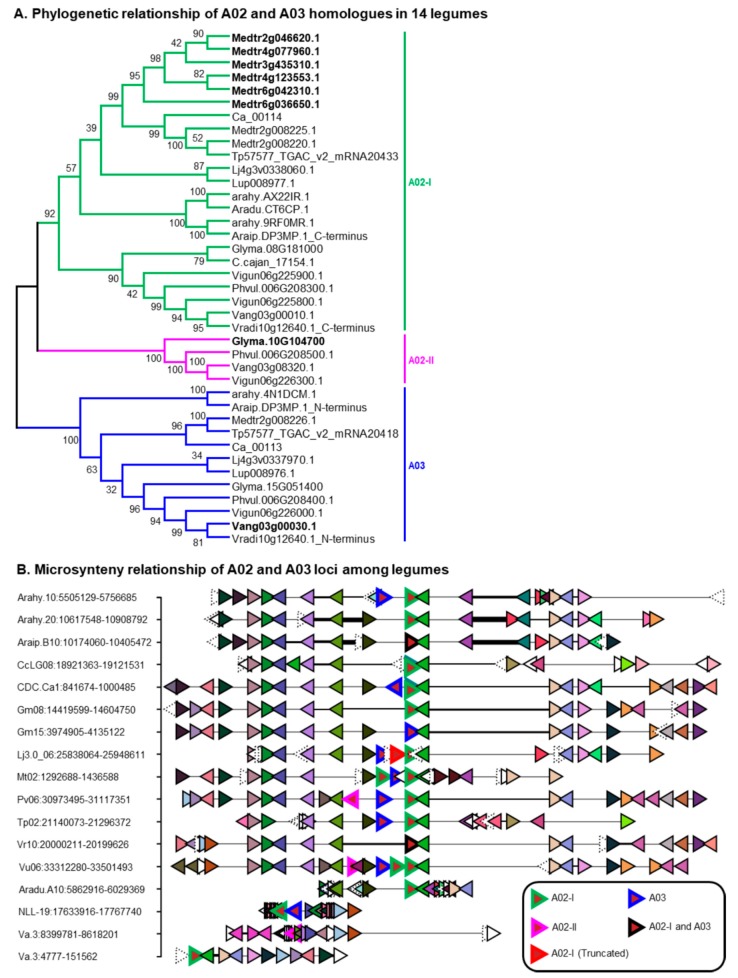
Evolutionary history of putative ortholog loci (POL) A02 and A03 in legumes. (**A**). Phylogenetic relationship of A02 and A03 homologs in 14 legumes. Bolded genes are non-synteny homologs with any of the 14 legumes. The full-length sequences of *Araip.DP3MP.1* and *Vradi10g12640.1* could be a sequencing error; their N-terminus and C-terminus shared high amino acid percent identity with *UGT91H5* (A02) and *UGT91H4* (A03) members respectively. See Table S9 for species and gene ID’s information. (**B**). Microsynteny relationship of A02 and A03 loci across legumes. Microsyntenic genome segments are retrieved and centered using *Phvul.006G208300*. Orthologous/paralogous gene pairs are indicated through the use of a common color. Uncolored and cracked genes are singletons and orphans respectively in this genomic region. Species and genomic positions are mentioned in the left side of each segment. From top to bottom, Arahy—*Arachis hypogea*, Araip—*Arachis ipaensis*, CcLG—*Cajanus cajan*, CDC.Ca—*Cicer arietinum*, Gm—*Glycine max*, Lj—*Lotus japonicus*, Mt—*Medicago truncatula*, Pv—*Phaseolus vulgaris*, Tp—*Trifolium pratense*, Vr—*Vigna radiata*, Vu—*Vigna unguiculate*, Aradu—*Arachis duranensis*, NLL—*Lupinus angustifolius*, and Va—*Vigna angularis*.

The two members [*Glyma.08G181000*: *UGT91H4* and *Glyma.10g104700*: *UGT91H9* (Figure 2A)] in A02 of *G. max* catalyze the addition of rhamnose or glucose, respectively, at the terminal position of C-3 sugar chain of SA and SB in vitro and in vivo [28,31]. The members of A02 from 14 legumes (Table S9) formed two sister clades (i.e., A02-I and A02-II) with high bootstrap support in phylogenetic analysis (Figure 2A). The A02-I locus corresponding the homologs of *UGT91H4* was located in syntenic blocks across all legumes and had one or two homologs in all the analyzed legumes except *M. truncatula,* which had two synteny and six non-synteny homologs (Figure 2B). Whereas, the A02-II locus corresponding *UGT91H9* homologs had single homologs only in millettoid species (e.g., *G. max*, *P. vulgaris,* and Vigna species) (Table S9) and found in syntenic blocks only between *P. vulgaris* and cow pea (*Vigna unguiculate*). The A02-I and A02-II members shared high amino acid identity (>70%) and showed a segmental duplication relationship in *G. max* and adzuki bean (*Vigna angularis*) and a tandem duplication relationship in *P. vulgaris* and cowpea (Figure 2B). Divergence time analysis estimates the duplication (whether it was tandem or segmental) may have occurred at ~44–47 MYA (Table S10). The eight UGTs in A02 of *M. truncatula* shared 71–90% amino acid identity and appear to be resulted from tandem as well as segmental duplication events (Table S8). None of these eight UGTs were characterized to date. The expression of a tandem duplicate *Medtr2g008220* and *Medtr2g008225* (*UGT91H6*) [both shared 90% amino acid identity; duplication time estimated as ~10.1 MYA (Table S10)] was highly correlated with triterpene biosynthetic genes [52]. Also, they shared 77% and 72% amino acid identity respectively to *UGT91H4* and found together with it in syntenic blocks (Figure 2B) suggesting that one of these two or both genes might have similar functions to that of *UGT91H*4.

A03 was a single-copy POL across all legumes and none of its members have been functionally characterized. Noteworthy, missense mutations in the PSPG box or its proximal regions of *Glyma.15g051400* (a A03 member of *G. max*) did not affect the soyasaponin profile [53]. The A03 member in *M. truncatula* (*Medtr2g008226*: *UGT91H5*) showed high co-expression values with triterpene biosynthetic genes [52] and tightly linked with a tandem duplicate of A02-I members (i.e., *Medtr2g008220* and *Medtr2g008225*), implying that A03 members may glycosylate triterpenes. Microsynteny analysis revealed that A03 locus tandemly linked to A02-I locus in diverse species including the early diverged legumes blue lupin (*Lupinus angustifolius*) and cultivated peanut (*Arachis hypogea*) (Figure 2B). Divergence time analysis estimates the tandem duplication event may have occurred ~42–84 MYA (Table S10).

**Figure 3:**
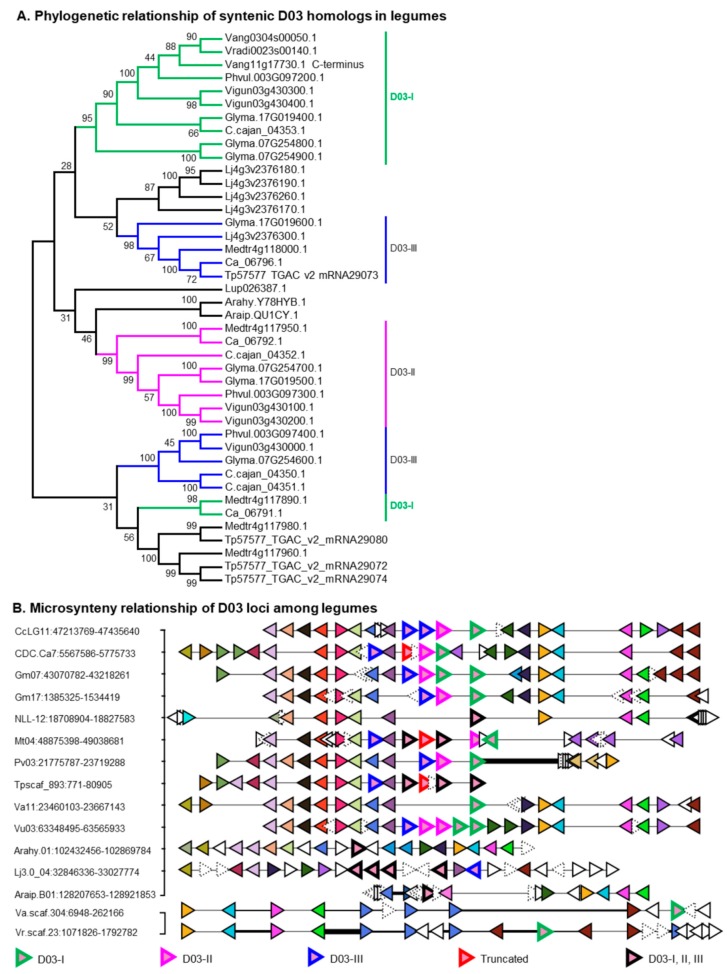
Evolutionary history of putative ortholog locus (POL) D03 in legumes. (**A**). Phylogenetic relationship of syntenic D03 homologs in 14 legumes. The full-length sequence of Vang11g17730.1 (1283 amino acids length) could be a sequencing error; only its C-terminus (471 amino acids) shared high amino acid percent identity with D03 members. Based on amino acid percent identity, D03 members are sub-grouped into D03-I, D03-II, and D03-III. Unresolved members are not sub-grouped. See Table S9 for species and gene id’s information. (**B**). Microsynteny relationship of D03 locus across legumes. Microsyntenic genome segments are retrieved and centered using Phvul.003G097300 and Araip.QU1CY in panel 1 and using Vang0304s00050 in panel 2. Orthologous/paralogous gene pairs are indicated through use of a common color. Uncolored and distorted genes are singletons and orphans respectively in this genomic region. Species and genomic positions are mentioned in the left side of each segment. From top to bottom, CcLG—*Cajanus cajan*, CDC.Ca—*Cicer arietinum*, Gm—*Glycine max*, NLL—*Lupinus angustifolius*, Mt—*Medicago truncatula*, Pv—*Phaseolus vulgaris*, Tp—*Trifolium pratense*, Va—*Vigna angularis*, Vu—*Vigna unguiculate*, Arahy—*Arachis hypogea*, Lj—*Lotus japonicus*, Araip—*Arachis ipaensis* and Vr—*Vigna radiata*.

D03 retained 17 members in *M. truncatula* and seven members in *G. max* (Table S9). One of these members from *M. truncatula* (*Medtr2g035020*: *UGT73F3*) was shown to glucosylate the C-28 position of hederagenin in vitro and in vivo [52]. Concurrently, a D03 member from *G. max* (*Glyma.07G254600*: *UGT73F2*) and its allelic variant *UGT73F4* were characterized to attach glucose or xylose respectively at the terminal position of C-22 sugar chain of SA in in vitro and in vivo [29]. These suggest that the catalytic functions of D03 members had been diverged and neofunctionalized during their course of evolution in species-specific manner. D03 existed as a multi-copy POL in hologalegina (eg. *M. truncatula*, *T. pratense* and *L. japonicus*) and millettoid species (eg. *G. max* and *P. vulgaris*) but a single-copy POL in the early diverged legumes (Table S9). Many of D03 members of *M. truncatula* and *T. pratense* were non-synteny homologs (Table S9) and showed a complex phylogenetic relationship (Figure S3). Even the syntenic D03 homologs from 14 legumes did not resolve well phylogenetically; however, they were divided into D03-I, D03-II, and D03-III clades based on the amino acid percent identity of D03 members (Figure 3A). The 17 MtUGTs in D03 shared 61–84% amino acid percent identity and may have resulted from more than one segmental and tandem duplication events (Table S8). *UGT73F3* shared 81–84% amino acid identity with its neighboring UGTs *Medtr2g034990* and *Medtr2g035040*, suggesting that these three UGTs may have resulted from a tandem duplication event occurred at ~11–16 MYA (Table S10). *UGT73F2* was tandemly located with three UGTs (*Glyma.07G254700*, *Glyma.07G254800,* and *Glyma.07G254900*) and all these showed high amino acid identity with another tandem duplicates located at the 17th chromosome (*Glyma.17G019400*, *Glyma.17G019500,* and *Glyma.17G019600*) suggesting that one of these genes first underwent tandem duplication and then copied into another chromosome by segmental duplication or GWGD. This notion is supported well by the gene-collinearity between Ch07 and Ch17 (Figure 3B). Divergence time analysis in *M. truncatula*, *G. max*, *P. vulgaris,* and chickpea (*Cicer arietinum*) estimates that the tandem duplication may have occurred ~40–104 MYA (Table S10). Non-sense mutations in *Glyma.07G254700*, *Glyma.07G254900*, *Glyma.17G019400*; *Glyma.17G019500* and *Glyma.17G019600* does not affect the saponin composition in mature soybean seeds implying that these genes might be not involved in soyasaponin biosynthesis [53]. The *Glyma.07G254800* was assumed as a pseudogene because the gene was not amplified using different primer sets [53].

D05 was a single-copy POL (Table S6). Its member (*Medtr4g031800*: *UGT73K1*) in *M. truncatula* reported to glycosylate hederagenin, SB, and soyasapogenol E in vitro [54]. Recently, the member of D05 (*Glyma.16G033700*) in *G. max* was reported to attach DDMP moieties at the C-22 hydroxyl position of SB in vivo [32]. In another independent study, we have identified that the D05 members are DDMP transferases and have homologs in diverse legume species including the early diverged ones [53]. These suggest that *UGT73K1* could be a candidate gene for DDMP transferase in *M. truncatula*. Noteworthy, *Glyma.16G033700* has been wrongly named as *UGT73B4* in Sundaramoorthy et al. [32].

**Figure 4:**
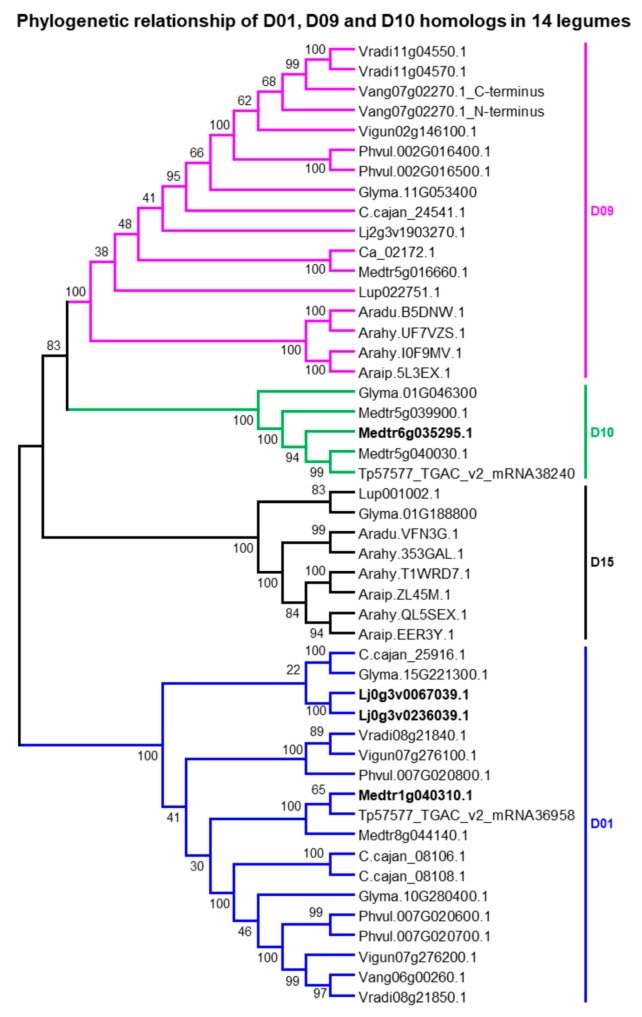
Phylogenetic relationship of putative ortholog loci (POL) D01, D09, and D10 homologs in 14 legumes. The full-length sequence of Vang07g02270.1 (901 amino acids length) could be a sequencing error; its C-terminus (409 amino acids) and N-terminus (492 amino acids) both shared high amino acid percent identity with D09 members. Bolded genes are non-synteny homologs with any of the 14 legumes. See Table S9 for species and gene ID’s information.

**Figure 5:**
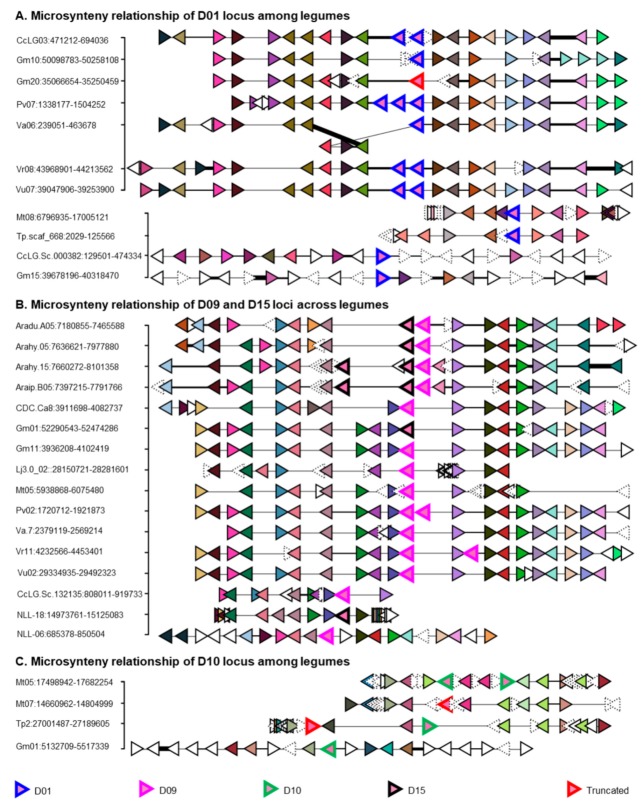
Microsynteny relationship of putative ortholog loci (POL) D01, D09, D10, and D15 in 14 legumes. (**A**). Microsyntenic genome segments having D01 locus are retrieved and centered using *Phvul.007G020600* (first panel) and *Medtr8g044140* (second panel). (**B**). Microsyntenic genome segments having D09 and D15 loci are retrieved and centered using Phvul.002G016400. (**C**). Microsyntenic genome segments having D10 locus are retrieved and centered using *tripr.gene37002* (Tp57577_TGAC_v2_mRNA38240). A–C, Orthologous/paralogous gene pairs are indicated through use of a common color. Uncolored and distorted genes are singletons and orphans respectively in these genome regions. Species and genomic positions are mentioned in the left side of each segment. Aradu—*Arachis duranensis*, Arahy—*Arachis hypogea*, Araip—*Arachis ipaensis*, CDC.Ca—*Cicer arietinum*, CcLG—*Cajanus cajan*, Gm—*Glycine max*, Lj—*Lotus japonicus*, NLL—*Lupinus angustifolius*, Mt—*Medicago truncatula*, Pv—*Phaseolus vulgaris*, Tp—*Trifolium pratense*, Va—*Vigna angularis*, Vr—*Vigna radiata*, and Vu—*Vigna unguiculate*. See Table S9 for species and gene ID’s information.

D09 (*Glyma.11G053400*: *UGT73P2*) and D10 (*Glyma.01g046300*: *UGT73P10*) members of *G. max* catalyze the addition of galactose and arabinose sugars respectively at the second position of the C-3 sugar chain of SB in vitro and/or in vivo [28,30]. The members of D01, D09, D10, and D15 from 14 legumes shared considerable amino acid identity and formed sister clades in phylogenetic analysis with high bootstrap support (Figure 1 and Figure 4). This implies that these four POLs may share a common ancestor and that the members of D01 and D15 may have a potential for triterpene glycosylation like that of D09/D10. Supporting this assumption, (i) a D01 member from *M. truncatula* (*Medtr8g044140*: *UGT73P1*) had been proposed to be involved in triterpene glycosylation because of its elevated co-expression with that of other triterpene biosynthetic genes upon methyl jasmonic acid treatment in *M. truncatula* root cell suspension cultures [54], and (ii) D15 members were tandemly linked to D09 in blue lupin and Arachis species (Figure 5). The presence/absence of D01, D09, D10, and D15 homologs in 14 legumes (Table S9) denote D09 was evolutionarily old and conserved, D15 was evolutionarily old but lost in many legumes and D01/D10 may have originated (via segmental duplication) after the PWGD but before the split of hologalegina and millettoid species (i.e., <59–48 MYA). However, assuming D01/D10/D15 were stemmed from D09, the divergence time analysis estimated that they were duplicated from D09 at 38−100, 54−81, and 73−101 MYA, respectively.

*G. max* retained single copy each for D09, D10, and D15 (Table S6). *M. truncatula* retained one copy for D09, three copies for D10, and none for D15. The D09 of *M. truncatula* (*Medtr5g016660*) shared 75.6% amino acid identity to *UGT73P2* and co-expressed highly with soyasaponin biosynthesis genes (Table S11) implying that *Medtr5g016660* might have similar functions to that of *UGT73P2*. The three D10 sequences in *M. truncatula* shared 74−75% amino acid identity and appear to be raised from tandem (*Medtr5g039900* and *Medtr5g040030*) and segmental duplication (*Medtr6g035295*) events occurred at ~15−18 MYA (Table S10). None of these genes were studied previously. However, based on the syntenic relationship and amino acid identity, we assume *Medtr5g039900* and *Medtr5g040030* were the most probable candidates to carry out the similar functions of *UGT73P10*. Both *G. max* and *M. truncatula* retained two copies in D01 that may have segmentally duplicated from one another at ~58 and ~11 MYA respectively (Table S10). Nonsense mutations in D01 (i.e., *Glyma.10G280400* and *Glyma.15G221300*) and D15 (*Glyma.01G188800*) members of *G. max* does not affect the soyasaponin composition [53] implying that these genes might be not involved in soyasaponin biosynthesis. The in vivo activity of UGT73P2 was never characterized before. We thus identified missense mutations causing various amino acid changes in PSPG box or its proximal region of *UGT73P2* but none of them affected the soyasaponin composition [22]. We reckon that the in vivo characterization of *UGT73P2* is essential to validate its function.

D18 of *G. max* included two members (*Glyma.08G348500* and *Glyma.08G348600*). One of these members (*Glyma.08G348500*) was co-expressed highly with *UGT73F2* and characterized to attach arabinose at the C-22-*O* position of SB-glycoside in vitro [23]. The resulting product was subsequently utilized by *UGT73F2* which attaches glucose as the second sugar to the C22-*O*-arabinose of SB in in vitro. Intriguingly, the products generated neither by *Glyma.08G348500* nor by *Glyma.08G348500*+*UGT73F2* are never identified in vivo in *G. max*. In this context, we reckon that *Glyma.08G348500* may not carry out its in vitro function in in vivo and it may arabinosylate the C-22-*O* position of SA or SA-glycosides in vivo in *G. max*. *Glyma.08G348500* and *Glyma.08G348600* were tandem duplicates sharing 63% amino acid identity (Table S8) and may have originated from one another at ~49 MYA (Table S10). D18 had one member each in *P. vulgaris*, Vigna species and in *T. subterraneum* but none in many other legumes (e.g., *M. truncatula*, *L. japonicus* and chickpea) (Table S6) implying that D18 may have undergone deletion process in those species.

D21 had homologs only in *T. pratense* and *T. subterraneum*. The relevance of D21 members in triterpene glycosylation yet to be discovered.

E06 of *M. truncatula* retained two members (*Medtr5g070040* and *Medtr5g070090*). One of these members (*Medtr5g070090*: *UGT71G1*) was proposed to be specific to medicagenic acid based on the integrated transcript and metabolite profiling in methyl jasmonic acid-treated *M. truncatula* root cell suspension cultures [54]. Since E06 was an only POL from group E as triterpene related and could glycosylate flavones and isoflavones with higher efficiency than triterpenes in in vitro [54], the in vivo function of *UGT71G1* or its homologs is necessitated to unarguably consider E06 as a triterpene related POL. *Medtr5g070040* and *Medtr5g070090* were tandem duplicates sharing 77% amino acid identity (diverged at ~14 MYA). In case of *G. max*, E06 retained three members resulted from tandem duplication (*Glyma.02G225800* and *Glyma.02G226000*) and segmental duplication/ GWGD (*Glyma.14G192800*) events.

## 3. Discussion

Hundreds of putative UGT sequences have been uncovered from the whole-genome sequence of several plant species including legumes (Table 1). All these studies have reported the species-specific expansion of UGT members in each phylogenetic group based on the identified UGT gene numbers within the phylogenetic groups among species. Albeit not without merits, these studies are never being sufficient enough to completely uncover the expansion, gene gain/ loss, and intron addition/deletion histories of UGTs. Identifying the putative ortholog groups across species and putative paralog groups within species is a valid and promising approach to estimate the mode of gene family expansion to determine gene functional differentiation to trace gene gain/loss events across species and to transfer functional information of well-studied genes from one species to non-studied species [55,56,57]. Previously, 24 ortholog groups were proposed using the UGTs from primitive and higher plant species and provided an overview like that of the phylogenetic group analysis [9]. However, assigning POLs to trace back all the identified UGTs from diverse species into a common ancestor and establishing their one-to-one, one-to-many, and many-to-many relationship across species are challenging and often jeopardized by the presence of multiple non-similar duplication events among the species. As a primary step, we thus herein assigned POLs for legume UGTs based on the tree-based (i.e., multi-species phylogenetic relationship) and graph-based (i.e., amino acid identity percentage) strategies to unravel the expansion pattern of UGT family and to decipher how triterpene UGTs evolved over the period in legumes.

### 3.1. Expansionary and Evolutionary Dynamics of the UGT Gene Family in M. truncatula and G. max: Insights from POL Assignments

Since legumes experienced different WGD events, it is mandatory to trace back each UGT genes from different legume genomes to a common ancestor that would help to deepen our knowledge on the evolutionary histories of UGTs in legumes. Hence, the 834 UGTs of all five legumes were traced back to 98 POLs. Since we were uncertain about the quality and completeness of genome assembly in *P. vulgaris*, *L. japonicus,* and *T. pratense*, we utilized POL assignment to determine the evolution and expansion patterns of UGT gene family in *M. truncatula* and *G. max*. Also, we wanted to clarify how *M. truncatula* retained most number of UGTs (*n* = 243) than its close relative *T. pratense*, both of which diverged around 23 MYA [58] and how *G. max* retained lesser UGTs (*n* = 208) than *M. truncatula* despite the recent glycine-specific WGD event. POL assignments clearly showed that, despite the high number of UGTs, *M. truncatula* lost 21 POLs during its course of evolution whereas *G. max* lost only 12. This suggests the higher/ lower number of UGTs in one species not necessarily correspond to that of the increased/decreased POLs. A simple comparison of POL numbers in other three legumes (*P. vulgaris*, *L. japonicus* and *T. pratense*) suggests that losing UGT POLs may be insubstantial but most legumes (if not all) would experience POL loss events either in species- or lineage-specific manner.

To pinpoint the expansion and mode of expansion, we subcategorized UGT POLs based on the gene copy number into single-, double-, and multi-copy POLs. *M. truncatula* and *G. max* retained respectively 33 and 40 single-copy POLs, 14 and 21 double-copy POLs, and 29 and 25 multi-copy POLS. Close observation of POLs revealed four key factors: (i) groups D and E contained the most number of UGTs in both species like other plant species (Table 1); but they still retained 5–10 single-copy POLs in those groups, (ii) expansion of group G was *M. truncatula*-specific but POL assignment showed that only G02 underwent rampant expansion while G01 and G04 carried each single UGT, (iii) expansion of group I was specific to *G. max*/*P. vulgaris* lineage but only I02 and I03 POLs expanded more whereas I01 and I04 POLs carried each single UGT, and (iv) eight single-copy POLs in *G. max* were expanded more in *M. truncatula* with 44 UGTs (~five-fold increase) while only two single copy POLs in *M. truncatula* were contained 3–4 UGTs in *G. max*. These results emphasize the fact that the increase in UGT number in *M. truncatula* was mostly achieved by POL-specific expansion and such POLs may be regarded as duplication susceptible. Despite the expansion, some POLs are tended to be duplication resistance (i.e., the single-copy POLs) in both species and such POLs may carry out important functions in plant growth, development, and protection. Nevertheless, current study predicts 86.4% [210 (149 tandem and 61 segmental) were duplicates in 243] of MtUGTs and 81.7% [170 (107 tandem and 63 segmental) were duplicates in 208] of GmUGTs were duplicates (Table S8). These sequences showed different degree of sequence conservation suggesting that they may have undergone rapid Ka and Ks nucleotide substitutions after the duplication event and retained for sub-or neo-functionalization.

More than 60% of UGTs in *M. truncatula* and 50% of UGTs in *G. max* were identified as tandem duplicates and formed cluster on different chromosomes suggesting that unequal crossover accelerated and contributed more in the expansion of UGT gene family in those species. Notably, the POLs G02 and D06 could respectively formed a cluster with 20 and 13 UGTs tandemly on chromosomes 6 and 8 in *M. truncatula*. Whereas, in *G. max*, the largest tandem cluster was formed with only six members (E10 and E13). Many tandem duplicates of GmUGTs found in synteny blocks of two corresponding chromosomes (e.g., A01, D03, and I02) suggesting that the segmental duplication or GWGD event also provided considerable contribution in the expansion of UGT gene family in *G. max*.

Intron addition/deletion events are a part of gene evolution. Previous studies (e.g., Li et al. [43]) mostly examined the intron addition/deletion histories of UGTs based on the mapping of introns positions. These studies provide information about the conserved intron position and approximate intron addition/deletion events. However, this information does not clarify intron deletion in no-intron UGTs or intron addition in one-intron UGTs. In this context, in this study, the UGT POLs were subcategorized into three types namely no-intron, one-intron, and multi-intron POLs (Table S7). This analysis showed that ~12% of no-intron UGTs in *M. truncatula* and *G. max* underwent intron addition and became one-intron UGTs during evolution. Although we could not detect any intron deletion events, POL assignments defined six phylogenetic groups (G, H, I, J, N, and P) as one-intron UGTs and two groups (O and R) as no-intron UGTs in legumes. Further results from diverse plant species are necessitated to determine whether this phenomenon is universal among higher plants. Nevertheless, this information will undoubtedly assist future studies to trace the intron addition/deletion history of UGTs in diverse species.

### 3.2. Evolutionary Insights into the Sugar Chain Biosynthesis of Soyasaponins

**Figure 6:**
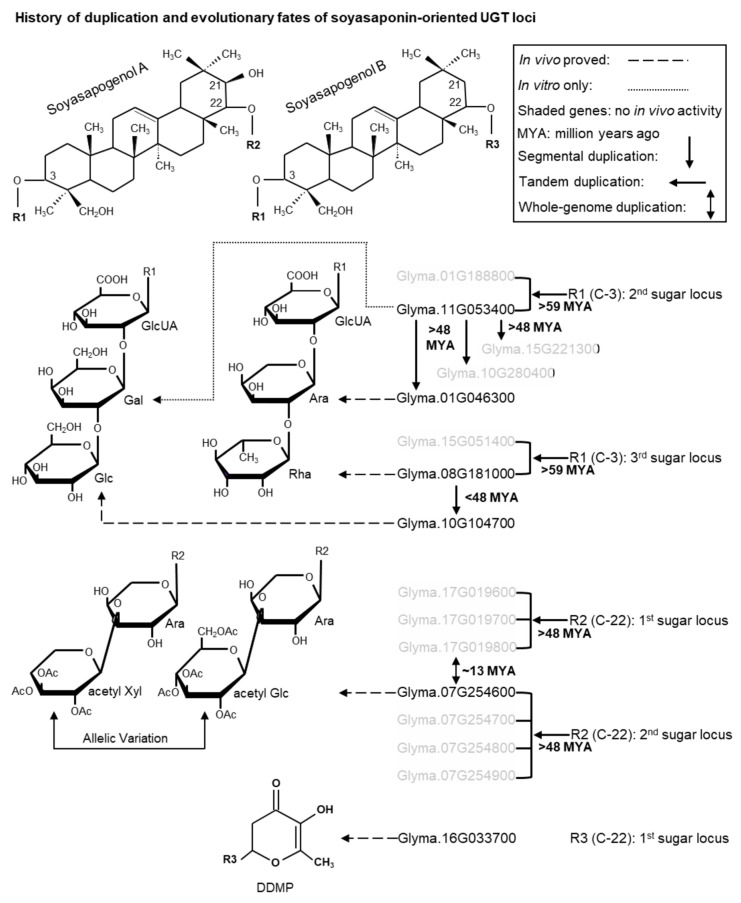
Duplication history and evolutionary fates of soyasaponin-related UGT loci. From top to bottom, structures of soyasapogenols A and B, major C-3 sugar chains, C-22 sugar chains and DDMP sugars are shown. Soyasaponin-related UGTs with their duplication history are shown on the right side of sugar chains. Divergence period of each duplication events is inferred based on the presence of corresponding homologs in other legumes (see Discussion Section 3.2.1 and Section 3.2.2). Shaded genes are not involved in soyasaponin biosynthesis in vivo [53]. UGTs are connected to responsible sugars by modified arrows: the enzymatic activity of dashed arrow UGTs is proved by in vivo experiments while the activity of round-dot arrow UGT is proved only by in vitro experiments. *Glyma.16G033700* may correspond a single-copy POL (D05) and its closest neighbor is the multi-copy POL D03. No UGTs have been characterized for the C-3-*O*-glycosylation of SA/SB and C-22-*O*-glycosylation of SA to-date.

The contribution of gene duplication followed by neofunctionalization (i.e., positive selection) is evident in the diversification of several groups of specialized metabolites [59] including triterpenoids [60]. However, no solid examples are currently available to emphasize the importance of gene duplication and neofunctionalization in the UGTs-oriented diversification of triterpenoids. Hence, in this study, we tried to establish the history and consequence of duplication on soyasaponin UGTs in triterpene glycosylation (Figure 6).

To reconstruct the evolutionary history of soyasaponin UGTs, it is mandatory to consider not only the gene duplication events but also the prevalence of soyasaponins in *G. max* and other legumes. Soyasaponins comprising bidesmosidic SA-glycosides (also known as group A saponins) and monodesmosidic SB-glycosides (also known as DDMP saponins) are predominantly accumulated in the seeds of *G. max*. Although many soyasaponin components identified in *G. max*, majority of them accumulated much lesser quantity in vivo and only the Aa/Ab and βg components correspond the maximum proportion of total group A and DDMP saponins respectively [22]. DDMP saponins and its derivatives group B or group E saponins are widespread among legumes while group A saponins are restricted to the subgenus *Soja* that includes the *G. max* and its wild relative *Glycine soja* (Gs).

#### 3.2.1. Evolution of the C-3 Sugar Chain of Soyasaponins

Six genuine C-3 sugar chains (four are tri-saccharide and two are di-saccharide) comprising of five sugars (glucuronic acid as first; galactose or arabinose as second; and rhamnose or glucose as third) are identified from soyasaponins (Figure 6). DDMP saponins having galactose (catalyzed by *UGT73P2*) as second sugar in the C-3 sugar chain (i.e., either of αg¸ βg and γg) are identified in many papilionoid legumes including the early diverged ones [e.g., cladrastis (*Styphnolobium japonicum*), genistoid (e.g., blue lupin) and dalbergioid (peanut) species] while those having arabinose (catalyzed by *UGT73P10*) at the same position (i.e., either of αa¸ βa, and γa) are exclusively reported in millettoid species (e.g., Phaseoleae and Desmodium species) except for *Amorpha fruticosa* (a dalbergioid species) [61]. Also, the homologs of *UGT73P2* (POL D09) were identified in syntenic blocks across legumes whereas *UGT73P10* homologs (POL D10) were identified in hologalegina and millettoid species (Figure 5; Table S9). These suggest that galactose being present at second position in the C-3 sugar chain catalyzed by *UGT73P2* is evolutionarily old and conserved. Since the specificity of *UGT73P10* towards soyasaponins was relatively lesser than *UGT73P2* (because all major soyasaponin components (i.e., Aa/Ab and βg) had only galactose as second sugar) and the loss of *UGT73P10* homologs was prevalent in many species (Table S9), we suspect *UGT73P10* must have stemmed out from *UGT73P2* and underwent gene deletion in many species but retained for neofunctionalization in some species especially in the millettoid lineage. Divergence time analysis estimates that the segmental duplication of *UGT73P2* may have occurred at ~54–81 MYA (i.e., before the PWGD) (Table S10). This coincides with the presence of βa in *A. fruticose* [61]. However, the identification of either of αa¸ βa, and γa, and the true homologs of *UGT73P10* in several early diverged legume species will clarify whether the duplication event occurred before the papilionoid speciation. Until then, based on the presence or absence of *UGT73P10* homologs in 14 legumes, we tentatively assume *UGT73P10* may have duplicated from *UGT73P*2 at >48 MYA (i.e., before the hologalegina-millettoid split) (Figure 6).

Like the galactose of C-3 sugar chain, the widespread occurrence of βg indicates rhamnose (catalyzed by *UGT91H4*) being present at the third position was evolutionarily old and conserved, whereas the restricted occurrence of αg and αa in legumes [61] indicates that glucose (catalyzed by *UGT91H9*) at the same position was evolutionarily recent. Notably, the homologs of *UGT91H4* (POL A02–clade I) were identified in syntenic blocks across legumes whereas *UGT91H9* homologs (POL A02–clade II) were identified only in millettoid species (Figure 2B; Table S9). These UGTs showed a segmental or tandem duplication relationship in the millettoid species and the duplication event may have occurred ~44–47 MYA (i.e., after the hologalegina–millettoid split). To support this notion, soyasaponins having glucose at the third position were never identified in legumes other than the millettoid species [61]. Though group A and DDMP saponins have glucose as third sugar in their C-3 sugar chain, only group A saponins (i.e., Aa and Ab) accumulated in high concentration in vivo while none of the DDMP saponins with glucose at the same position accumulated predominantly. Considering these facts, we assume *UGT91H9* must have stemmed out from *UGT91H4* and followed neofunctionalization with high specificity towards group A saponins (Figure 6).

#### 3.2.2. Evolution of the C-22 Sugar Chain of Soyasaponins

In addition to the C-21 hydroxyl position, group A and DDMP saponins are mainly differenced at the C-22-*O* position of their aglycones where the former has arabinose while the latter has DDMP (Figure 6). Distribution of soyasaponins among legumes [61] suggests DDMP moiety at the C-22-*O* position of soyasapogenols (catalyzed by *UGT73K’s* [32,53]; POL D05) is evolutionarily old and conserved. The members of D05 shared considerable amino acid identity to D03 members and clustered neighborly (Figure 1) suggesting that both were evolutionarily and phylogenetically related. POL D03 of *G. max* retained a set of tandem duplicated genes in two different chromosomes (i.e., Ch07 and Ch17). One of the genes from the sets was characterized for the addition of second (xylose/glucose catalyzed by *UGT73F4*/*UGT73F6*; from Ch07) sugar of the C-22 sugar chain of group A saponins (Figure 6). We believe the identification of a gene responsible for the C-22-*O*-arabinosylation will shed more lights on the biosynthetic origin of group A saponins. Of note, though SA identified in other than glycine species (e.g., *M. truncatula* [62] and lupine [63]), group A saponins were only identified in glycine species. Notably, lupine accumulates SA with general C-3 sugar chain (i.e., Rha-Gal-GlcUA-) but had only one sugar at the C-22-*O* position and that too xylose not arabinose [63]. These suggest the gene of C-22-*O*-arabinosylation may have evolved by species-specific functional divergence.

### 3.3. Triterpene Related UGT POLs and Their Functional Divergence

UGTs modulate the functionalities of different triterpene aglycones by glycosylating them at various active sites depending on the genetic background of given genera/species. Intriguingly, the discovery of UGTs for specific triterpenes in legumes is scarce. For example, the legume model plant *M. truncatula* accumulates at least ten different triterpene aglycones including medicagenic acid, hederagenin, and soyasapogenols, attached with various hexose sugars at various active sites [62]; yet, only three UGTs have been characterized for triterpenes (Table S1). To accelerate/ease the search of UGTs of beneficial triterpenoids in legumes, we herein utilized our POL assignments to estimate the candidate UGTs for legumes triterpenes.

To narrow down triterpene-related UGTs in legumes, we first collected all the studied UGTs of *M. truncatula*, *G. max*, *L. japonicus*, *P. vulgaris,* and *T. pratense* from published literature and mapped the information in the POL tree. UGTs of 15 in *M. truncatula*, 34 in *G. max*, 7 in *L. japonicus* and 1 in *P. vulgaris* have been studied so far; of these, 3 MtUGTs, 8 GmUGTs and 1 LjUGT are characterized for triterpene glycosylation (Tables S1–S5). These 12 UGTs were evolutionarily related to 11 POLs (Figure 1). Earlier studies show that a part of group A, D and E members of legume UGTs are capable to glycosylate triterpenes (Figure 1). Since none of group E members of legumes characterized for triterpene glycosylation in vivo and *UGT71G1* (only this member was attributed as triterpene related and belonged to POL E06) glycosylated flavones and isoflavones with higher efficiency than triterpenes in in vitro [54], we believe that group E members shall not be specific for triterpenes. We also underline that researchers shall not conclude the functions of a given UGT solely based on the in vitro experiments, without its or its homologs in vivo functional analysis. This notion could be supported by several examples. To describe few, (i) *UGT73K1* glucosylated hederagenin, SB and soyasapogenol E in vitro [54] but its homologs attached DDMP moieties to SB in vivo [32,48), and (ii) *UGT73F2* glycosylated isoflavones in vitro [64] but it was later reported to be specific for soyasaponins using in vivo and in vitro experiments [29].

Based on the phylogenetic clustering of characterized UGTs, we propose here that at least four POLs from group A (A02, A03, A09, and A10) and 13 POLs from group D (D01, D03, D05–D10, D15, D16, D18, D19, and D21) could glycosylate diverse triterpene scaffolds in different legume species. During our study to discover soyasaponin UGTs in *G. max*, we found that the members of A03 (*Glyma.15G051400*), D01 (*Glyma.10G280400* and *Glyma.15G221300*), D03 (*Glyma.07G254700*–*Glyma.07G254900* and *Glyma.17G019400*–*Glyma.17G019600*), D08 (*Glyma.02G104600*), and D15 (*Glyma.01G188800*) are not involved in soyasaponin biosynthesis in vivo and one of them glycosylated either of soyasaponin aglycones or glycosides in vitro [53]. In this context, we presume that the homologs of these genes in other species may glycosylate diverse triterpenes in vitro and/or in vivo. Supporting this notion, a homolog of D08 in *Glycyrrhiza uralensis* (*GuUGAT*; GenBank ID: ANJ03631.1–79.3% amino acid identity to *Glyma.02G104600*) [65] glycosylated C-3-*O* position of glycyrrhetinic acid in vitro and a homolog of D03 namely *UGT73F17* (GenBank ID: AXS75258.1–69.4% amino acid identity to *Glyma.17G019500*) from *G. uralensis* glycosylated C-30 of glycyrrhizic acid [66]. Though the in vivo functions of *GuUGAT* and *UGT73F17* remain to be studied, these data imply D03 and D08 members may underwent species-specific functional divergence.

## 4. Materials and Methods

### 4.1. Identification of Putative UGTs in Five Legumes

Proteins containing the PF00201 (UDP-glucuronosyl/glucosyltransferase) domain were retrieved for *M. truncatula*, *G. max*, *P. vulgaris*, *L. japonicus,* and *T. pratense* from the LIS database (http://legumeinfo.org/) [51]. Concurrently, a stand-alone blast-p search was performed with the PSPG sequence of known UGTs (*UGT73F3* for *M. truncatula*, *L. japonicus*, *T. pratense* and *UGT73F2* for *G. max* and *P. vulgaris*) against the available respective proteome data in Phytozome v12.1 database (https://phytozome.jgi.doe.gov/pz/portal.html#) [50] and Miyakogusa database (http://www.kazusa.or.jp/lotus; for *L. japonicus*) [67]. These databases were searched with default settings except the function ‘# the number of alignments to show’ in Phytozome that was set at 300. All the retrieved primary sequences (i.e., spliced transcripts ignored) were manually checked; and, proteins that had incomplete PSPG compare to their orthologs/paralogs and proteins whose first amino acid is not methionine were excluded from the study. Additionally, short-length proteins (i.e., proteins having less than 350 amino acids), and too lengthy proteins (i.e., proteins having more than 600 amino acids) were excluded. If UGTs of same species share 100% amino acid identity at full-length protein level, one of UGTs from the given identical pair was excluded further. Criteria of each excluded sequence from this study were described in Tables S1–S5.

### 4.2. Phylogenetic Analysis

To determine the evolutionary relationship and the presence/absence of UGT’s phylogenetic groups, the selected amino acid sequences of five legume species were aligned together or separately with 14 AtUGTs (groups A-N), three ZmUGTs (groups O-Q), and one CsUGT (group R) by MUSCLE and used to construct neighbor-joining (NJ)-oriented unrooted phylogenetic trees. All multiple sequence alignments and phylogenetic trees generation were performed by MEGA6 program [68]. Trees were constructed under Poisson model, uniform rates and pairwise deletion options with 1000 bootstrap replicates which values were expressed as percentages in each node.

### 4.3. Assignment of POL for Legume UGTs

To assign UGTs POL, multi-species phylogenetic trees were constructed separately for each phylogenetic group in MEGA6 (Figures S1–S11). Trees were generated as mentioned in the previous section. A POL was assigned based on phylogenetic clustering and the amino acid percent identity of full-length proteins, by applying two conditions that sequence conservation shall be relatively high among UGTs in the given POL across species and the UGTs must cluster together in the phylogenetic analyses. Sequence identities were inferred from Clustal Omega (http://www.ebi.ac.uk/Tools/msa/clustalo/) [69]. POLs were named within each group in chronological order according to MtUGTs present in the given POL. If no MtUGTs are present in the given POL, the next species UGTs positions were utilized for the POL naming.

### 4.4. Estimation of Intron Addition or Deletion Events

To estimate intron addition or deletion events, first, intron information of all the identified UGTs was mapped in the POL assignments data (Table S7). Second, POLs were classified into three types namely (i) no-intron POLs, (ii) one-intron POLs, and (iii) mixed-intron POLs, based on the intron numbers present in the given POL across species. If most (if not all) members from three or more species in the given POL sustain no or one intron, that given POL was defined as no-intron POL or one-intron POL, respectively. Mixed-intron POLs sustain members with or without introns, which could not let us to make any concrete decision. Third, intron addition or deletion events were examined for no-intron and one-intron POLs: if the members do not follow the designation of a given POL, they were considered as intron-gained or intron-lost members. Additionally, if any members from any intron POL types had more than one intron, they were considered as intron gained members.

### 4.5. Chromosomal Mapping, Gene Duplication, and Divergence Time Analyses

The physical locations of UGTs were plotted on chromosomes by Map Chart 2.2 software [70] using the chromosomal coordinates of MtUGTs and GmUGTs that were respectively inferred from their most recent genome versions. UGT members in each POL within species could be considered as paralogs and across species could be considered as orthologs. The term homologs represent both paralogs and orthologs within and across species. Duplicated copies separated by four or fewer other gens were attributed as tandem duplicates while other copies were attributed as segmental duplicates.

The amino acid sequences of each duplicated pair or each duplicated group were aligned separately by MUSCLE in MEGAX [71] using neighbor-joining method, with the first and/or final 10 amino acids in each alignment were checked manually and modified if necessary. These alignments were then used to guide the alignment of corresponding coding sequences in RevTrans 2.0b server [72]. The resulting coding sequence alignments were utilized for the calculation of synonymous (dS) and nonsynonymous (dN) nucleotide substitution rates per site using yn00 tool implemented in PAML package [73]. The obtained dS values of Nei-Gojobori method were used in the formula T = dS/ (2 × λ) × 10^−6^ to estimate the divergence time (T) of duplicated pairs. Assuming the PWGD occurred at ~58 MYA, the λ (rate of dS nucleotide substitutions per site per year) was 1.08 × 10^−8^, 5.85 × 10^−9^, 8.46 × 10^−9^, 6.05 × 10^−9^ and 8.12 × 10^−9^ for *M. truncatula* [74], *G. max* [75], *P. vulgaris* [75], *C. arietinum* [76] and *A. duranensis*/*A. ipaensis* [77] species, respectively.

### 4.6. Microsynteny Analyses

Microsynteny relationship of triterpenoid-related UGTs among legumes was inferred from the online tool Genome Context Viewer (https://legumeinfo.org/lis_context_viewer/instructions) [78]. Gene IDs of *G. max*, *M. truncatula* or *P. vulgaris* belonged to triterpenoid-related POLs were subjected and the tool was run with default settings. The resulting output files were aligned using MS office.

## 5. Conclusions

Based on the multi-species phylogenetic relationship and amino acid identity percentage, POLs were successfully assigned to each UGTs identified in this study. The loss/retention of POLs, addition/deletion of introns and the multiplication of UGTs in a given POL were merely species-specific followed by lineage-specific. Notably, a rampant duplication in four POLs accounted for 30% of total UGTs in *M. truncatula* while that never happened for other legumes. In *M. truncatula* and *G. max*, 43–47% of POLs retained single copies and the remaining of them retained two or multiple copies accounting 80–85% of the total number of UGTs. Tandem duplication majorly contributed to the expansion of UGT family in *M. truncatula* (61.3%) and *G. max* (51.4%). Besides the expansion, both species lost many UGTs and different POLs in species-specific manner during their course of evolution. UGTs reported to diversify the C-3 sugar chain of soyasaponins were all resulted from two independent duplication events while the UGTs reported for the C-22-*O* glycosylation of soyasaponins were evolutionarily close. The members from 13 group D and 4 group A POLs could be triterpene related. In sum, our study paved a way to decipher evolutionary dynamics of UGTs, emphasized the contribution of duplication and neofunctionalization of UGTs in triterpene glycoside diversification and will assist in precise selection of candidate UGTs for various specialized metabolites across legumes.

## Figures and Tables

**Table 1:** Number of plant UGTs in different phylogenetic groups.

No.	Plant Species Name *	No. UGTs in Different Phylogenetic Groups																		Total UGTs	Ref.
		A	B	C	D	E	F	G	H	I	J	K	L	M	N	O	P	Q	R		
1	*Mimulus guttatus*	10	2	3	11	14	–	12	1	4	2	9	17	2	1	9	3	–	–	100	[36]
2	*Camellia sinensis*	15	5	2	20	23	2	13	2	2	2	1	27	3	–	6	6	–	3	132	[37]
3	*Vitis vinifera*	23	3	4	8	46	5	15	7	14	4	2	31	5	1	2	11	–	–	181	[36]
4	*Linum usitatissimum*	16	5	6	21	22	1	19	6	9	4	5	19	3	1	–	–	–	–	137	[38]
5	*Populus trichocarpa*	12	2	6	14	49	–	42	5	5	6	2	23	6	1	3	2	–	–	178	[36]
6	*Cucumis sativus*	10	1	2	12	13	–	11	5	–	2	1	17	2	1	3	5	–	–	85	[36]
7	*Arabidopsis thaliana*	14	3	3	13	22	3	6	19	1	2	2	17	1	1	–	–	–	–	107	[36]
8	*Brassica rapa*	12	4	4	24	31	1	9	18	1	3	3	26	2	2	–	–	–	–	140	[39]
9	*Brassica napus*	17	10	10	36	48	2	14	35	2	3	6	61	4	3	–	–	–	–	251	[39]
10	*Brassica oleraca*	15	7	4	23	32	–	8	23	1	2	3	32	2	2	–	–	–	–	154	[39]
11	*Cajanus Cajan*	2	2	1	36	33	–	9	–	5	–	–	12	2	–	6	12	–	–	120	[40]
12	*Glycine max*	25	3	1	43	36	1	15	3	18	3	2	19	4	1	5	3	–	–	182	[36]
	*Glycine max*	5	1	2	38	46	6	16	2	4	–	–	18	5	–	6	–	–	–	149	[41]
	*Glycine max*	21	3	–	46	52	8	16	3	17	7	–	19	5	1	6	4	–	–	208	This study
13	*Phaseolus vulgaris*	19	3	2	33	33	5	18	3	15	3	–	17	4	1	6	5	–	1	168	This study
14	*Lotus japonicus*	9	3	–	25	22	2	9	1	2	1	0	10	1	1	6	1	–	1	94	This study
15	*Medicago truncatula*	28	4	–	55	55	2	39	3	5	9	–	33	2	1	3	3	–	1	243	This study
16	*Trifolium pratense*	11	3	–	29	39	1	13	3	1	2	–	12	1	–	2	3	–	1	121	This study
17	*Malus domestica*	33	4	7	13	55	6	40	14	11	12	6	16	13	1	5	5	–	–	241	[36]
18	*Prunus mume*	16	2	3	17	23	3	18	10	4	?	8	17	3	?	–	–	–	–	130	[42]
19	*Prunus persica*	10	2	4	19	29	4	34	9	5	7	7	18	14	1	1	4	–	–	168	[43]
20	*Oryza sativa*	14	9	8	26	38	–	20	7	9	3	1	23	5	2	6	9	–	–	180	[36]
21	*Triticum aestivum*	22	3	2	17	37	2	4	5	7	5	–	19	3	1	3	13	36	–	179	[44]
22	*Sorghum bicolor*	10	4	6	24	50	–	17	12	8	3	1	26	6	3	8	2	–	–	180	[36]
23	*Zea mays*	8	3	5	18	34	2	12	9	9	3	1	23	3	4	5	1	7	–	147	[45]

*—Species ordered in phylogenetic relevance; ‘–‘—UGTs not detected/absent in the respective species; ‘?’—Unknown in the corresponding paper.

**Table 2:** Distribution of UGT POLs among the legumes.

Phylogenetic Groups	Distribution of UGT POLs among the Phylogenetic Groups ^a^					
	*M. truncatula*	*G. max*	*P. vulgaris*	*L. japonicus*	*T. pratense*	Total
A	10 (28)	10 (21)	09 (19)	08 (09)	07 (11)	12
B	03 (04)	03 (03)	02 (03)	02 (03)	03 (03)	03
C	–	–	01 (02)	–	–	01
D	14 (55)	19 (46)	16 (33)	15 (25)	13 (29)	21
E	18 (55)	21 (52)	16 (33)	12 (22)	14 (39)	23
F	01 (02)	03 (08)	03 (05)	02 (02)	01 (01)	03
G	05 (39)	04 (16)	03 (18)	03 (09)	04 (13)	05
H	03 (03)	02 (03)	03 (03)	01 (01)	03 (03)	03
I	02 (05)	04 (17)	04 (15)	02 (02)	01 (01)	04
J	04 (09)	05 (07)	02 (03)	01 (01)	02 (02)	05
K	–	–	–	–	–	–
L	09 (33)	08 (19)	09 (17)	07 (10)	07 (12)	10
M	02 (02)	02 (05)	02 (04)	01 (01)	01 (01)	02
N	01 (01)	01 (01)	01 (01)	01 (01)	-	01
O	02 (03)	02 (06)	02 (06)	02 (06)	02 (02)	02
P	01 (03)	02 (04)	02 (05)	01 (01)	01 (03)	02
Q	–	–	–	–	–	–
R	01 (01)	–	01 (01)	01 (01)	01 (01)	01
Total	76 (243)	86 (208)	76 (168)	59 (94)	60 (121)	98

^a^ Numbers within the brackets denotes the number of UGTs corresponded to the loci.

## Supplementary Materials

Supplementary materials can be found at https://www.mdpi.com/1422-0067/21/5/1855/s1.

## References

[1] Coutinho P.M., Deleury E., Davies G.J., Henrissat B. (2003). An evolving hierarchical family classification for glycosyltransferases. J. Mol. Biol..

[2] Schuman B., Alfaro J.A., Evans S.V., Peters T. (2007). Glycosyltransferase structure and function. Topics in Current Chemistry.

[3] Wagner G.K., Pesnot T. (2010). Glycosyltransferases and their assays. ChemBioChem.

[4] Lairson L.L., Henrissat B., Davies G.J., Withers S.G. (2008). Glycosyltransferases: Structures, functions, and mechanisms. Annu. Rev. Biochem..

[5] Liang D.M., Liu J.H., Wu H., Wang B.B., Zhu H.J., Qiao J.J. (2015). Glycosyltransferases: Mechanisms and applications in natural product development. Chem. Soc. Rev..

[6] Osmani S.A., Bak S., Møller B.L. (2009). Substrate specificity of plant UDP-dependent glycosyltransferases predicted from crystal structures and homology modeling. Phytochemistry.

[7] Lombard V., Golaconda Ramulu H., Drula E., Coutinho P.M., Henrissat B. (2013). The carbohydrate-active enzymes database (CAZy) in 2013. Nucleic Acids Res..

[8] Bowles D., Lim E.K., Poppenberger B., Vaistij F.E. (2006). Glycosyltransferases of lipophilic small molecules. Annu. Rev. Plant Biol..

[9] Yonekura-Sakakibara K., Hanada K. (2011). An evolutionary view of functional diversity in family 1 glycosyltransferases. Plant J..

[10] Gachon C.M., Langlois-Meurinne M., Saindrenan P. (2005). Plant secondary metabolism glycosyltransferases: The emerging functional analysis. Trends Plant Sci..

[11] Wang X. (2009). Structure, mechanism and engineering of plant natural product glycosyltransferases. FEBS Lett..

[12] Bowles D., Isayenkova J., Lim E.K., Poppenberger B. (2005). Glycosyltransferases: Managers of small molecules. Curr. Opin. Plant Biol..

[13] Härtl K., MacGraphery K., Rüdiger J., Schwab W., Schwab W., Lange B.M., Wüst M. (2018). Tailoring natural products with glycosyltransferases. Biotechnology of Natural Products.

[14] Brazier-Hicks M., Offen W.A., Gershater M.C., Revett T.J., Lim E.K., Bowles D.J., Davies G.J., Edwards R. (2007). Characterization and engineering of the bifunctional *N*- and *O*-glucosyltransferase involved in xenobiotic metabolism in plants. Proc. Natl. Acad. Sci. USA.

[15] Li X., Michlmayr H., Schweiger W., Malachova A., Shin S., Huang Y., Dong Y., Wiesenberger G., McCormick S., Lemmens M. (2017). A barley UDP-glucosyltransferase inactivates nivalenol and provides Fusarium Head Blight resistance in transgenic wheat. J. Exp. Bot..

[16] Goossens A., Osbourn A., Michoux F., Bak S. (2018). Triterpene messages from the EU-FP7 project TriForC. Trends Plant Sci..

[17] Osbourn A., Goss R.J.M., Field R.A. (2011). The saponins—Polar isoprenoids with important and diverse activities. Nat. Prod. Rep..

[18] Seki H., Tamura K., Muranaka T. (2018). Plant-derived isoprenoid sweetners: Recent progress in biosynthetic gene discovery and perspectives on microbial production. Biosci. Biotech. Biochem..

[19] Thimmappa R., Geisler K., Louveau T., O’Maille P., Osbourn A. (2014). Triterpene biosynthesis in plants. Annu. Rev. Plant Biol..

[20] Salmon M., Thimmappa R.B., Minto R.E., Melton R.E., Hughes R.K., O’Maille P.E., Hemmings A.M., Osbourn A. (2016). A conserved amino acid residue critical for product and substrate specificity in plant triterpene synthases. Proc. Natl. Acad. Sci. USA.

[21] Xue Z., Duan L., Liu D., Guo J., Ge S., Dicks J., O’Maille P., Osbourn A., Qi X. (2012). Divergent evolution of oxidosqualene cyclases in plants. New Phytol..

[22] Krishnamurthy P., Fujisawa Y., Takahashi Y., Abe H., Yamane K., Mukaiyama K., Son H.R., Hiraga S., Kaga A., Anai T. (2019). High throughput screening and characterization of a high-density soybean mutant library elucidate the biosynthesis pathway of triterpenoid saponins. Plant Cell Physiol..

[23] Louveau T., Orme A., Pfalzgraf H., Stephenson M.J., Melton R., Saalbach G., Hemmings A.M., Leveau A., Rejzek M., Vickerstaff R.J. (2018). Analysis of two new arabinosyltransferases belonging to the carbohydrate-active enzyme (CAZY) glycosyl transferase family 1 provides insights into disease resistance and sugar donor specificity. Plant Cell.

[24] Augustin J.M., Drok S., Shinoda T., Sanmiya K., Nielsen J.K., Khakimov B., Olsen C.E., Hansen E.H., Kuzina V., Ekstrøm C.T. (2012). UDP-glycosyltransferases from the UGT73C subfamily in *Barbarea vulgaris* catalyse sapogenin 3-O-glucosylation in saponin-mediated insect resistance. Plant Physiol..

[25] Guang C., Chen J., Sang S., Cheng S. (2014). Biological functionality of soyasaponins and soyasapogenols. J. Agri. Food Chem..

[26] Rahman A., Tsurumi S. (2002). The unique auxin influx modulator chromosaponin I: A physiological overview. Plant Tissue Cult..

[27] Yano R., Takagi K., Takada Y., Mukaiyama K., Tsukamoto C., Sayama T., Kaga A., Anai T., Sawai S., Ohyama K. (2017). Metabolic switching of astringent and beneficial triterpenoid saponins in soybean is achieved by a loss-of-function mutation in cytochrome P450 72A69. Plant, J..

[28] Shibuya M., Nishimura K., Yasuyama N., Ebizuka Y. (2010). Identification and characterization of glycosyltransferases involved in the biosynthesis of soyasaponin I in *Glycine max*. FEBS Lett..

[29] Sayama T., Ono E., Takagi K., Takada Y., Horikawa M., Nakamoto Y., Hirose A., Sasama H., Ohashi M., Hasegawa H. (2012). The *Sg-1* glycosyltransferase locus regulates structural diversity of triterpenoid saponins of soybean. Plant Cell.

[30] Takagi K., Yano R., Tochigi S., Fujisawa Y., Tsuchinaga H., Takahashi Y., Takada Y., Kaga A., Anai T., Tsukamoto C. (2018). Genetic and functional characterization of Sg-4 glycosyltransferase involved in the formation of sugar chain structure at the C-3 position of soybean saponins. Phytochemistry.

[31] Yano R., Takagi K., Tochigi S., Fujisawa Y., Nomura Y., Tsuchinaga H., Takahashi Y., Takada Y., Kaga A., Anai T. (2018). Isolation and characterization of the soybean Sg-3 gene that is involved in genetic variation in sugar chain composition at the C-3 position in soyasaponins. Plant Cell Physiol..

[32] Sundaramoorthy J., Par G.T., Komagamine K., Tsukamoto C., Chang J.H., Lee J.D., Kim J.H., Seo H.S., Song J.T. (2019). Biosynthesis of DDMP saponins in soybean is regulated by a distinct UDP-glycosyltransferase. New Phytol..

[33] Christenhusz M.J.M., Byng J.W. (2016). The number of known plant species in the world and its annual increase. Phytotaxa.

[34] Wink M. (2013). Evolution of secondary metabolites in legumes (Fabaceae). S. Afr. J. Bot..

[35] Wang J., Sun P., Li Y., Liu Y., Yu J., Ma X., Sun S., Yang N., Xia R., Lei T. (2017). Hierarchically aligning 10 legume genomes establishes family-level genomics platform. Plant Physiol..

[36] Caputi L., Malnoy M., Goremykin V., Nikiforova S., Martens S. (2012). A genome-wide phylogenetic reconstruction of family 1 UDP-glycosyltransferases revealed the expansion of the family during the adaptation of plants to life on land. Plant J..

[37] Cui L., Yao S., Dai X., Yin Q., Liu Y., Jiang X., Wu Y., Qian Y., Pang Q., Gao L. (2016). Identification of UDP-glycosyltransferases involved in the biosynthesis of astringent taste compounds in tea (*Camellia sinensis*). J. Exp. Bot..

[38] Barvkar V.T., Pardeshi V.C., Kale S.M., Kadoo N.Y., Gupta V.S. (2012). Phylogenomic analysis of UDP glycosyltransferase 1 multigene family in *Linum usitatissimum* identified genes with varied expression patterns. BMC Genomics.

[39] Rehman H.M., Nawaz M.A., Shah Z.H., Ludwig-Muller J., Chung G., Ahmad M.Q., Yang S.H., Lee S.I. (2018). Comparative genomic and transcriptomic analyses of Family-1 UDP glycosyltransferase in three Brassica species and Arabidopsis indicates stress-responsive regulation. Sci. Rep..

[40] Song Z., Niu L., Yang Q., Dong B., Wang L., Dong M., Fan X., Jian Y., Meng D., Fu Y. (2019). Genome-wide identification and characterization of UGT family in pigeonpea (*Cajanus cajan*) and expression analysis in abiotic stress. Trees.

[41] Rehman H.M., Nawaz M.A., Bao L., Shah Z.H., Lee J.M., Ahmad M.Q., Chung G., Yang S.H. (2016). Genome-wide analysis of family-1 UDP-glycosyltransferases in soybean confirms their abundance and varied expression during seed development. J. Plant Physiol..

[42] Zhang Z., Zhuo X., Yan Z., Zhang Q. (2018). Comparative genomic and transcriptomic analyses of family-1 UDP glycosyltransferase in *Prunus mume*. Int. J. Mol. Sci..

[43] Wu B., Gao L., Gao J., Xu Y., Liu H., Cao X., Zhang B., Chen K. (2017). Genome-wide identification, expression patterns, and functional analysis of UDP glycosyltransferase family in peach (*Prunus persica* L. Batsch). Front. Plant Sci..

[44] He Y., Ahmad D., Zhang X., Zhang Y., Wu L., Jiang P., Ma H. (2018). Genome-wide analysis of family-1 UDP glycosyltransferases (UGT) and identification of UGT genes for FHB resistance in wheat (*Triticum aestivum* L.). BMC Plant Biol..

[45] Li Y., Li P., Wang Y., Dong R., Yu H., Hou B. (2014). Genome-wide identification and phylogenetic analysis of Family-1 UDP glycosyltransferases in maize (*Zea mays*). Planta.

[46] Yin Q., Shen G., Di S., Fan C., Chang Z., Pang Y. (2017). Genome-wide identification and functional characterization of UDP-glucosyltransferase genes involved in flavonoid biosynthesis in *Glycine max*. Plant Cell Physiol..

[47] Yin Q., Shen G., Chang Z., Tang Y., Gao H., Pang Y. (2017). Involvement of three putative glucosyltransferases from the UGT72 family in flavonol glucoside/rhamnoside biosynthesis in *Lotus japonicus* seeds. J. Exp. Bot..

[48] Tomcal M., Stiffler N., Barkan A. (2013). POGs2: A web portal to facilitate cross-species inferences about protein architecture and function in plants. PLoS ONE.

[49] Van Bel M., Diels T., Vancaester E., Kreft L., Botzki A., Van de Peer Y., Coppens F., Vandepoele K. (2017). PLAZA 4.0: An integrative resource for functional, evolutionary and comparative plant genomics. Nucleic Acids Res..

[50] Goodstein D.M., Shu S., Howson R., Neupane R., Hayes R.D., Fazo J., Mitros T., Dirks W., Hellsten U., Putnam N. (2012). Phytozome: A comparative platform for green plant genomics. Nucleic Acids Res..

[51] Dash S., Campbell J.D., Cannon E.K., Cleary A.M., Huang W., Kalberer S.R., Karingula V., Rice A.G., Singh J., Umale P.E. (2016). Legume information system (LegumeInfo.org): A key component of a set of federated data resources for the legume family. Nucleic Acids Res..

[52] Naoumkina M.A., Modolo L.V., Huhman D.V., Urbanczyk-Wochniak E., Tang Y., Sumner L.W., Dixon R.A. (2010). Genomic and coexpression analyses predict multiple genes involved in triterpene saponin biosynthesis in Medicago truncatula. Plant Cell.

[53] Ishimoto M’s Research Group. Identification of UGTs involved in soyasaponin glycosylation.

[54] Achnine L., Huhman D.V., Farag M.A., Sumner L.W., Blount J.W., Dixon R.A. (2005). Genomics-based selection and functional characterization of triterpene glycosyltransferases from the model legume Medicago truncatula. Plant J..

[55] Proost S., Van Bel M., Sterck L., Billiau K., Van Parys T., Van de Peer Y., Vandepoele K. (2009). PLAZA: A comparative genomics resource to study gene and genome evolution in plants. Plant Cell.

[56] Trachana K., Larsson T.A., Powell S., Chen W.H., Doerks T., Muller J., Bork P. (2011). Orthology prediction methods: A quality assessment using curated protein families. Bioessays.

[57] Walker N.S., Stiffler N., Barkan A. (2007). POGs/PlantRBPs: A resource for comparative genomics in plants. Nucl. Acid Res..

[58] De Vega J.J., Ayling S., Hegarty M., Kudrna D., Goicoechea J.L., Ergon A., Rognli O.A., Jones C., Swain M., Geurts R. (2015). Red clover (*Trifolium pretense* L.) draft genome provides a platform for trait improvement. Sci. Rep..

[59] Ober D. (2005). Seeing double: Gene duplication and diversification in plant secondary metabolism. Trends. Plant Sci..

[60] Hamberger B., Bak S. (2013). Plant P450s as versatile drivers for evolution of species-specific chemical diversity. Philos. Trans. R. Soc. B.

[61] Okubo K., Yoshiki Y., Waller G.R., Yamasaki K. (1996). Oxygen-radical-scavenging activity of DDMP-conjugated saponins and physiological role in leguminous plant. Saponins Used in Food and Agriculture.

[62] Pollier J., Morreel K., Geelen D., Goossens A. (2011). Metabolite profiling of triterpene saponins in Medicago truncatula hairy roots by liquid chromatography fourier transform ion cyclotron resonance mass spectrometry. J. Nat. Prod..

[63] Kinjo J., Kishida F., Watanabe K., Hashimoto F., Nohara T. (1994). Five new triterpene glycosides from Russell lupine. Chem. Parm. Bull..

[64] Dhaubhadel S., Farhangkhoee M., Chapman R. (2004). Identification and characterization of isoflavonoid specific glycosyltransferase and malonyltransferase from soybean seeds. J. Exp. Bot..

[65] Xu G., Cai W., Gao W., Liu C. (2016). A novel glucuronosyltransferase has an unprecedented ability to catalyse continuous two-step glucuronosylation of glycyrrhetinic acid to yield glycyrrhizin. New Phytol..

[66] He J., Chen K., Hu Z.M., Li K., Song W., Yu L.Y., Leung C.H., Ma D.L., Qiao X., Ye M. (2018). UGT73F17, a new glycosyltransferase from *Glycyrrhiza uralensis*, catalyzes the regiospecific glycosylation of pentacyclic triterpenoids. Chem. Commun..

[67] Sato S., Nakamura Y., Kaneko T., Asamizu E., Kato T., Nakao M., Sasamoto S., Watanabe A., Ono A., Kawashima K. (2008). Genome structure of the legume, *Lotus japonicus*. DNA Res..

[68] Tamura K., Stecher G., Peterson D., Filipski A., Kumar S. (2013). MEGA6: Molecular evolutionary genetics analysis version 6.0. Mol. Biol. Evol..

[69] Sievers F., Higgins D.G. (2014). Clustal omega. Curr. Protoc. Bioinform..

[70] Voorrips R.E. (2002). MapChart: Software for the graphical presentation of linkage maps and QTLs. J. Hered..

[71] Kumar S., Stecher G., Li M., Knyaz C., Tamura K. (2018). MEGA X: Molecular evolutionary genetics analysis across computing platforms. Mol. Biol. Evol..

[72] Wemersson R., Pedersen A.G. (2003). RevTrans-Constructing alignments of coding DNA from aligned amino acid sequences. Nucleic Acids Res..

[73] Yang Z. (2007). PAML 4: Phylogenetic analysis by maximum likelihood. Mol. Biol. Evol..

[74] Young N.D., Debelle F., Oldroyd G.E.D., Geurts R., Cannon S.B., Udvardi M.K., Benedito V.A., Mayer K.F.X., Gouzy J., Schoof H. (2011). The Medicago genome provides insight into the evolution of rhizobial symbioses. Nature.

[75] Schmutz J., McClean P.E., Mamidi S., Wu G.A., Cannon S.B., Grimwood J., Jenkins J., Shu S., Song Q., Chavarro C. (2014). A reference genome for common bean and genome-wide analysis of dual domestications. Nat. Genet..

[76] Jain M., Misra G., Patel R.K., Priya P., Jhanwar S., Khan A.W., Shah N., Singh V.K., Garg R., Jeena G. (2013). A draft genome sequence of the pulse crop chickpea (*Cicer arietinum* L.). Plant J..

[77] Bertioli D.J., Cannon S.B., Froenicke L., Huang G., Farmer A.D., Cannon E.K.S., Liu X., Gao D., Clevenger J., Dash S. (2016). The genome sequences of *Arachis duranensis* and *Arachis ipaensis*, the diploid ancestors of cultivated peanut. Nat. Genet..

[78] Cleary A., Farmer A. (2018). Genome Context Viewer: Visual exploration of multiple annotated genomes using microsynteny. Bioinformatics.

