# Supplementary material for: Reconstruction of the Evolutionary Histories of UGT Gene Superfamily in Legumes Clarifies the Functional Divergence of Duplicates in Specialized Metabolism
Source: Int J Mol Sci. 2020 Mar 8;21(5):1855. doi: 10.3390/ijms21051855 (PMC7084467; doi:10.3390/ijms21051855)
Supplement: Supplementary file 1 [file ijms-21-01855-s001.zip › ijms-713495_supllementary files/01. 20200227_Figs. S1-S14.pdf]

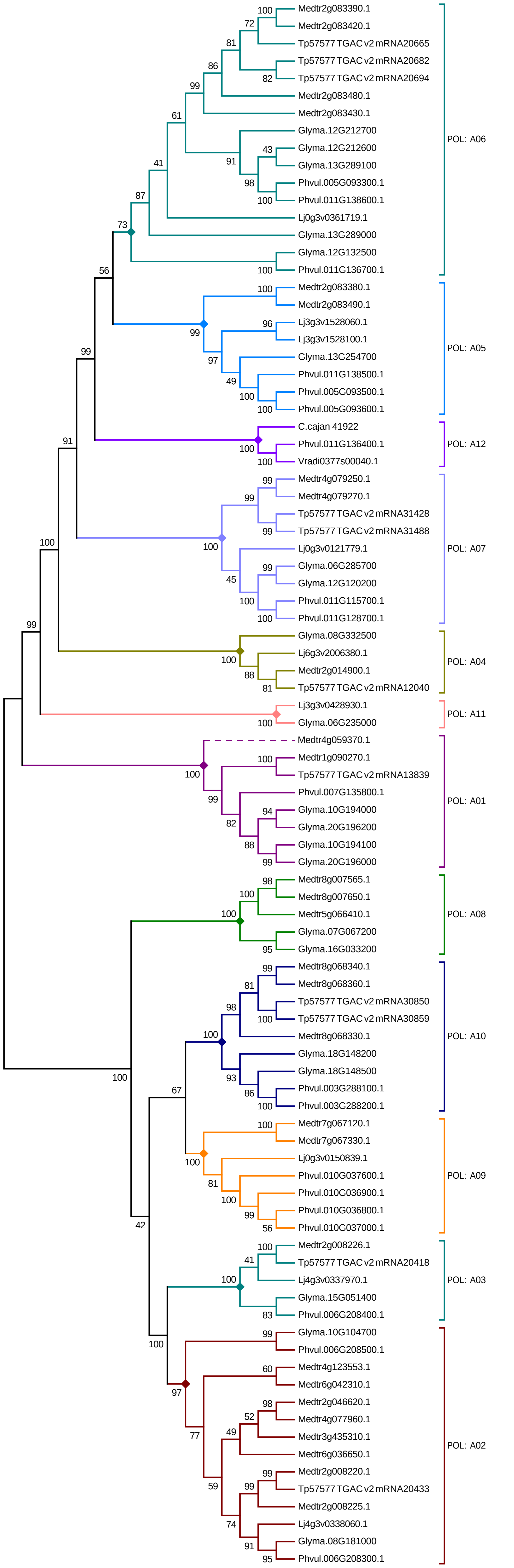

Fig. S1. Putative ortholog loci (POL) in Group A UGTs of Mt, Gm, Pv, Lj and Tp.
The optimal tree with the sum of branch length = 12.97704608 is shown.
- The analysis involved 90 amino acid sequences.
- All ambiguous positions were removed for each sequence pair.
- There were a total of 625 positions in the final dataset.
- Bootstrap percentages (1000 replicates) are shown next to the branches.
Ortholog groupings are shown in different color.
- Braked brach implies the gene may diverged or correspond a new POL.

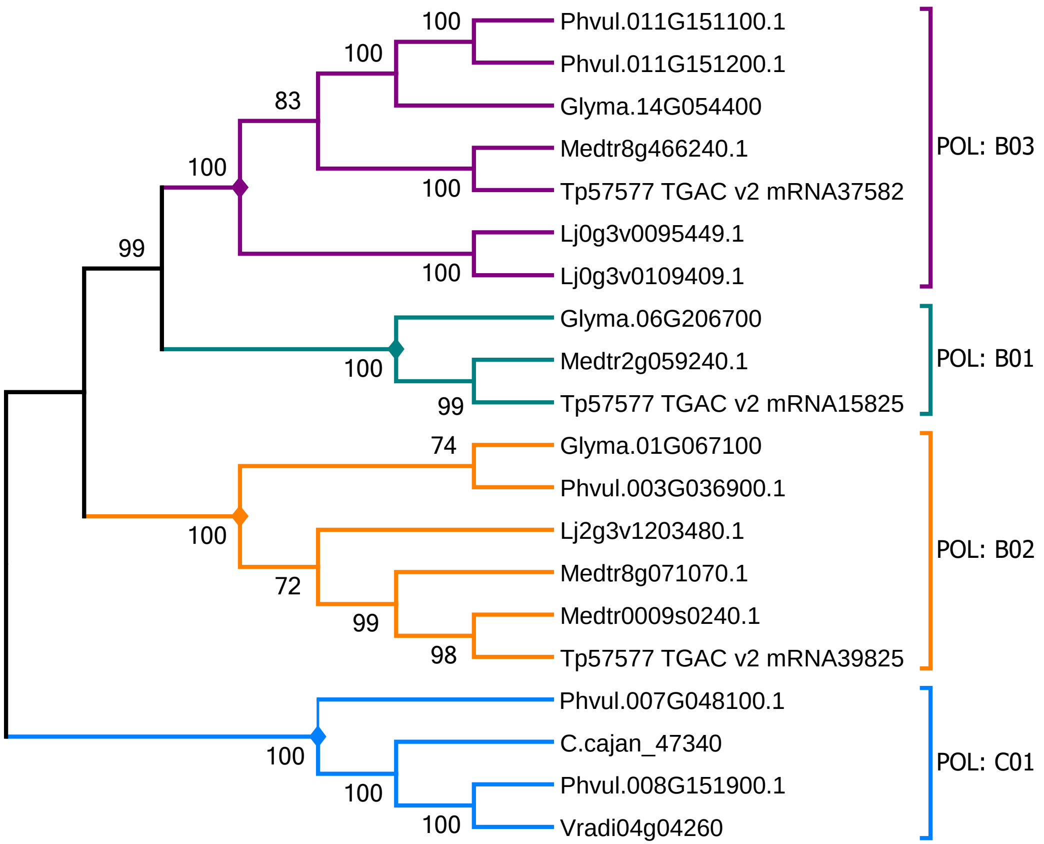

Fig. S2. Putative ortholog loci (POL) in Group B and C UGTs of *Mt*, *Gm*, *Pv*, *Lj*, and *Tp*.

The optimal tree with the sum of branch length = 4.21650156 is shown.

- The analysis involved 20 amino acid sequences.
- All ambiguous positions were removed for each sequence pair.
- There were a total of 538 positions in the final dataset.
- Bootstrap percentages (1000 replicates) are shown next to the branches.

Ortholog groupings are shown in different color.

- Braked branch implies the gene may diverged or correspond a new POL.
- Some UGTs are used from other legumes to validate the POLs.

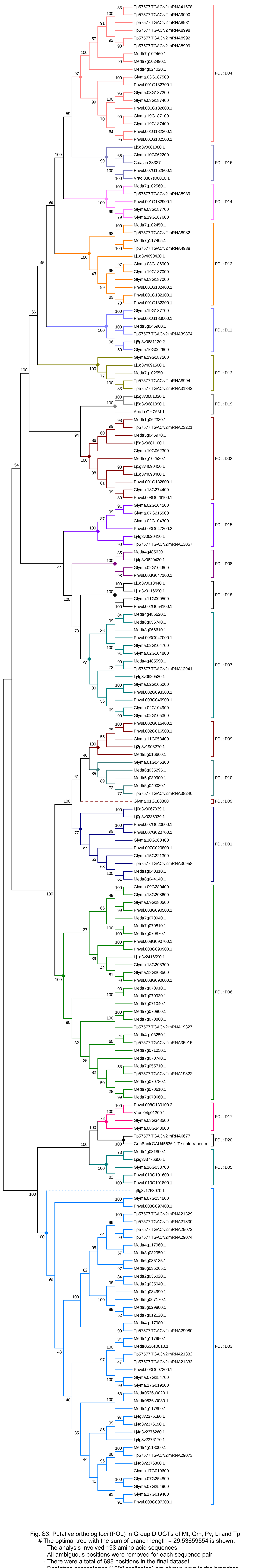

Fig. S3. Putative ortholog loci (POL) in Group D UGTs of Mt, Gm, Pv, Lj and Tp.

The optimal tree with the sum of branch length = 29.53659554 is shown.
- The analysis involved 193 amino acid sequences.
- All ambiguous positions were removed for each sequence pair.
- There were a total of 698 positions in the final dataset.
- Bootstrap percentages (1000 replicates) are shown next to the branches.

Ortholog groupings are shown in different color.
- Braked branch implies the gene may diverged or correspond a new POL.
- Some UGTs are used from other legumes to validate the POLs.

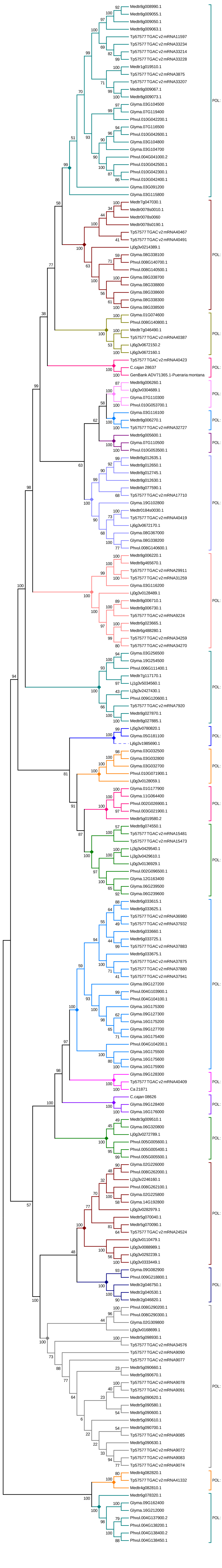

Fig. S4. Putative ortholog loci (POL) in Group E UGTs of Mt, Gm, Pv, Lj and Tp.
The optimal tree with the sum of branch length = 33.74015575 is shown.
- The analysis involved 205 amino acid sequences.
- All ambiguous positions were removed for each sequence pair.
- There were a total of 988 positions in the final dataset.
- Bootstrap percentages (1000 replicates) are shown next to the branches.
Orthologs groupings are shown in different color.
- Braked branch implies the gene may diverged or correspond a new POL.
- Some UGTs are used from other legumes to validate the POLs.

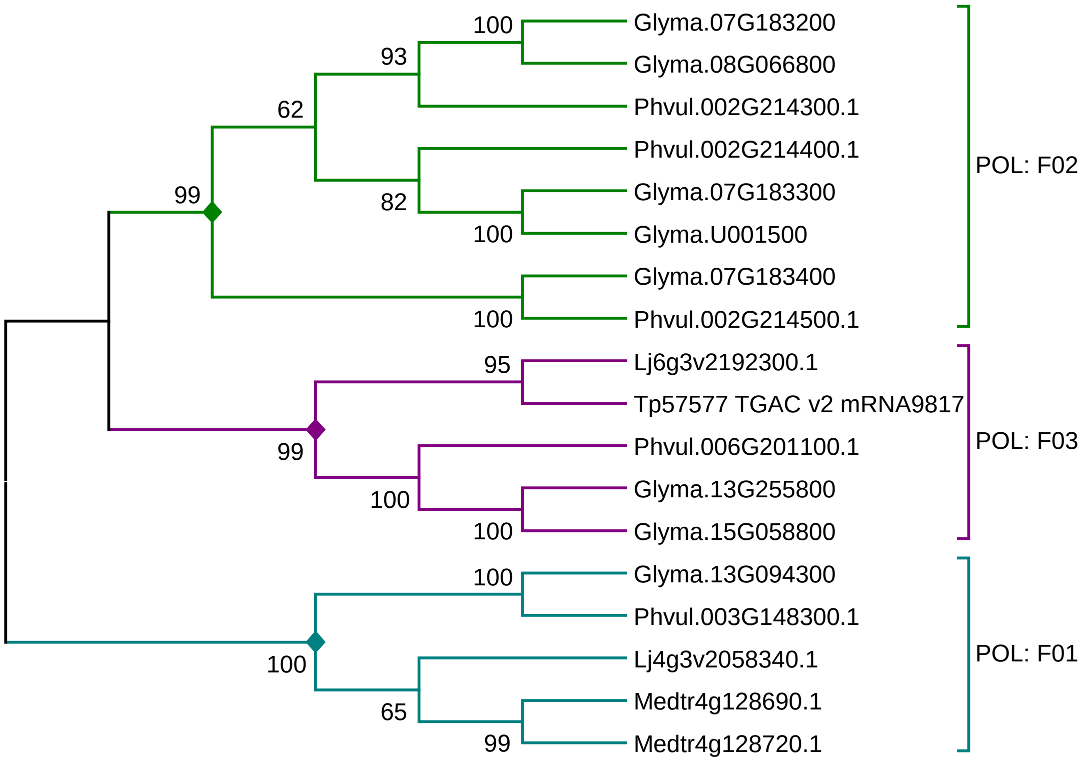

Fig. S5. Putative ortholog loci (POL) in Group F UGTs of *Mt*, *Gm*, *Pv*, *Lj*, and *Tp*.

The optimal tree with the sum of branch length = 3.13335182 is shown.

- The analysis involved 18 amino acid sequences.
- All ambiguous positions were removed for each sequence pair.
- There were a total of 515 positions in the final dataset.
- Bootstrap percentages (1000 replicates) are shown next to the branches.

Ortholog groupings are shown in different color.

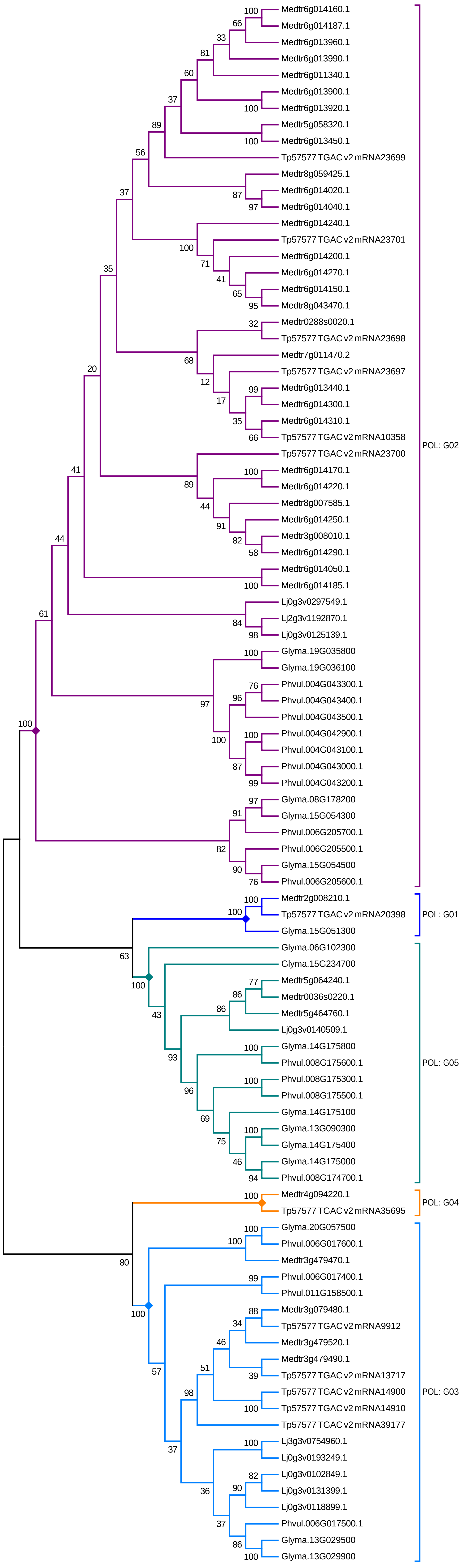

Fig. S6. Putative ortholog loci (POL) in Group G UGTs of Mt, Gm, Pv, Lj and Tp.
The optimal tree with the sum of branch length = 11.13934061 is shown.
- The analysis involved 95 amino acid sequences.
- All ambiguous positions were removed for each sequence pair.
- There were a total of 647 positions in the final dataset.
- Bootstrap percentages (1000 replicates) are shown next to the branches.
Ortholog groupings are shown in different color.

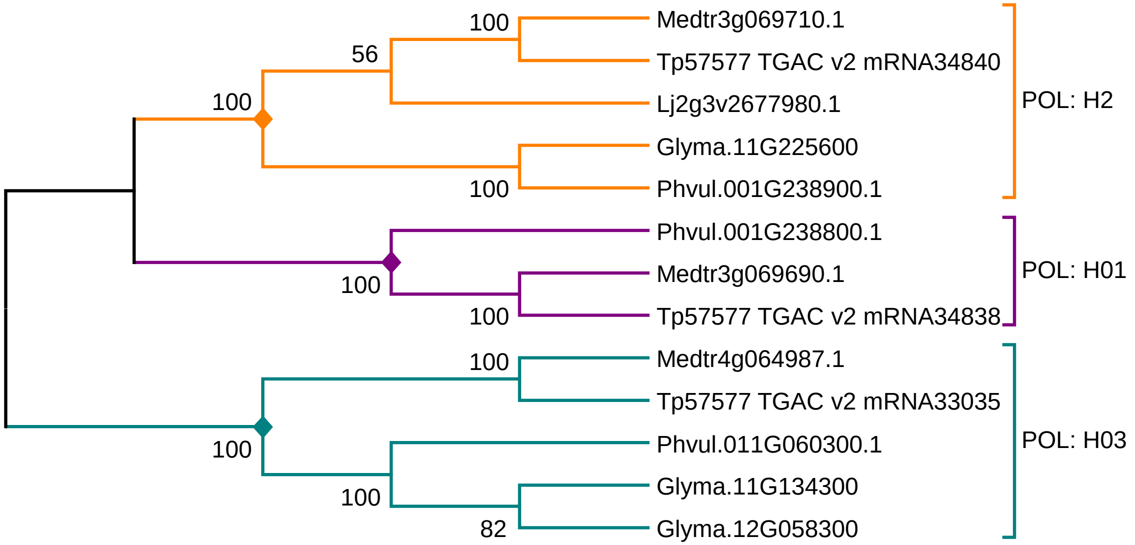

Fig. S7. Putative ortholog loci (POL) in Group H UGTs of *Mt*, *Gm*, *Pv*, *Lj*, and *Tp*.

The optimal tree with the sum of branch length = 2.10419870 is shown.

- The analysis involved 13 amino acid sequences.
- All ambiguous positions were removed for each sequence pair.
- There were a total of 508 positions in the final dataset.
- Bootstrap percentages (1000 replicates) are shown next to the branches.

Ortholog groupings are shown in different color.

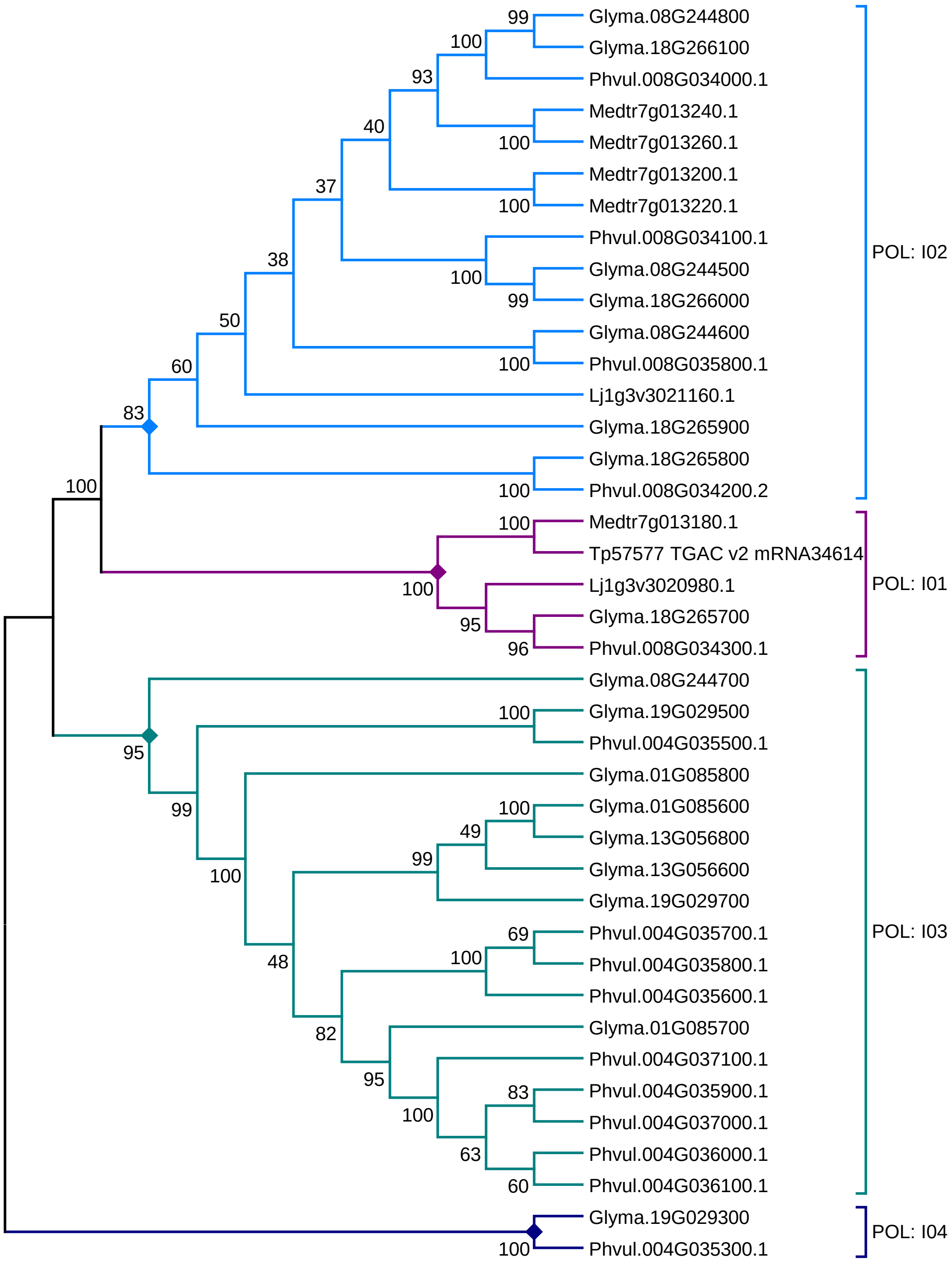

Fig. S8. Putative ortholog loci (POL) in Group I UGTs of *Mt*, *Gm*, *Pv*, *Lj*, and *Tp*.

The optimal tree with the sum of branch length = 5.49405989 is shown.

- The analysis involved 40 amino acid sequences.
- All ambiguous positions were removed for each sequence pair.
- There were a total of 505 positions in the final dataset.
- Bootstrap percentages (1000 replicates) are shown next to the branches.

Ortholog groupings are shown in different color.

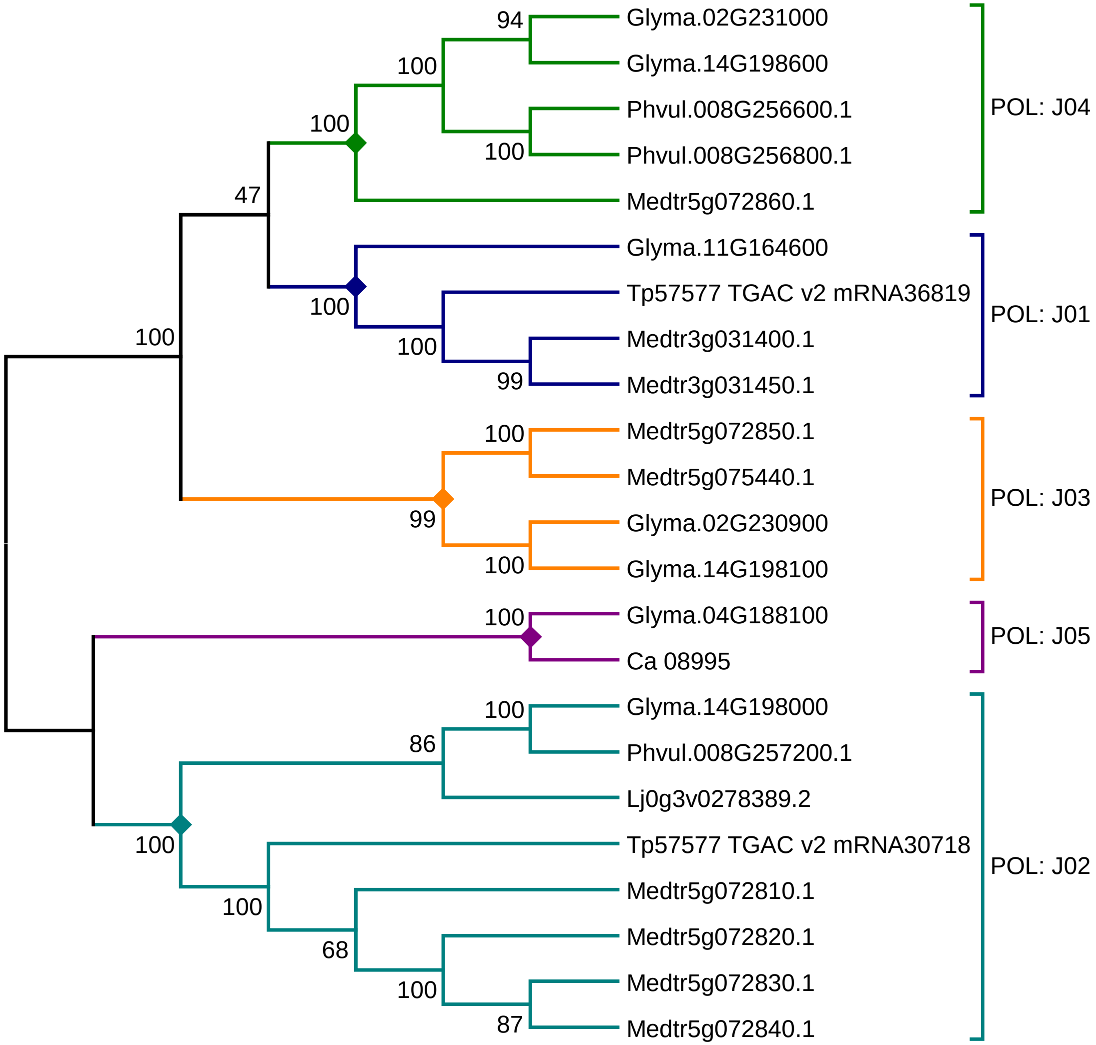

Fig. S9. Putative ortholog loci (POL) in Group J UGTs of *Mt*, *Gm*, *Pv*, *Lj*, and *Tp*.

The optimal tree with the sum of branch length = 3.74404269 is shown.

- The analysis involved 23 amino acid sequences.
- All ambiguous positions were removed for each sequence pair.
- There were a total of 555 positions in the final dataset.
- Bootstrap percentages (1000 replicates) are shown next to the branches.

Ortholog groupings are shown in different color.

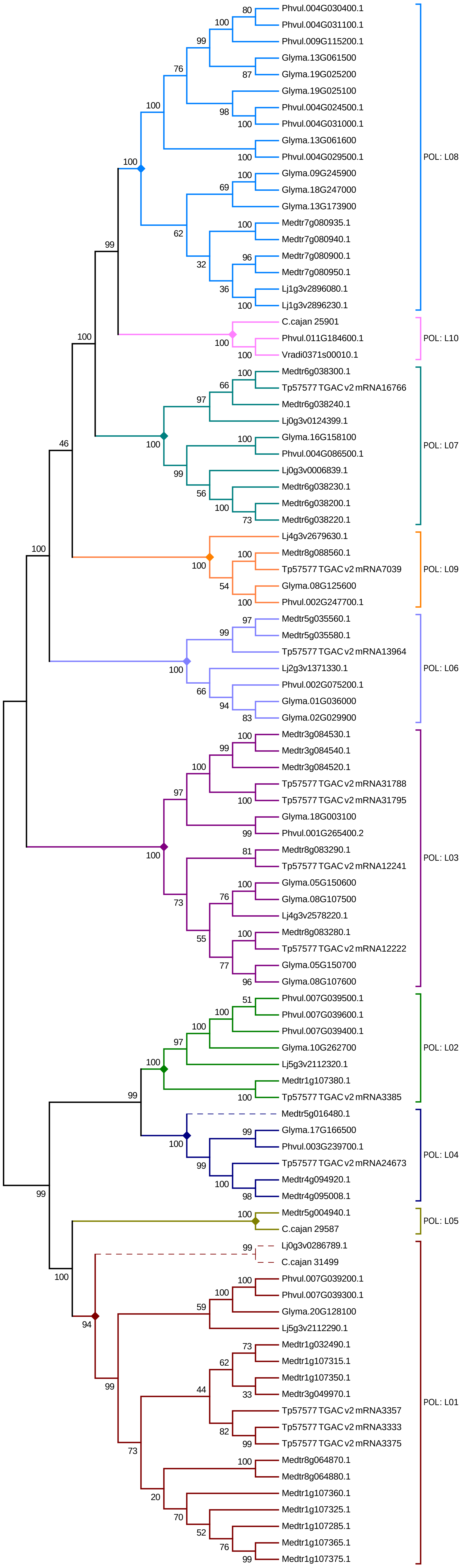

Fig. S10. Putative ortholog loci (POL) in Group L UGTs of Mt, Gm, Pv, Lj and Tp.
The optimal tree with the sum of branch length = 15.18292942 is shown.
- The analysis involved 95 amino acid sequences.
- All ambiguous positions were removed for each sequence pair.
- There were a total of 694 positions in the final dataset.
- Bootstrap percentages (1000 replicates) are shown next to the branches.
Ortholog groupings are shown in different color.
- Braked branches may diverged or correspond a new POL.

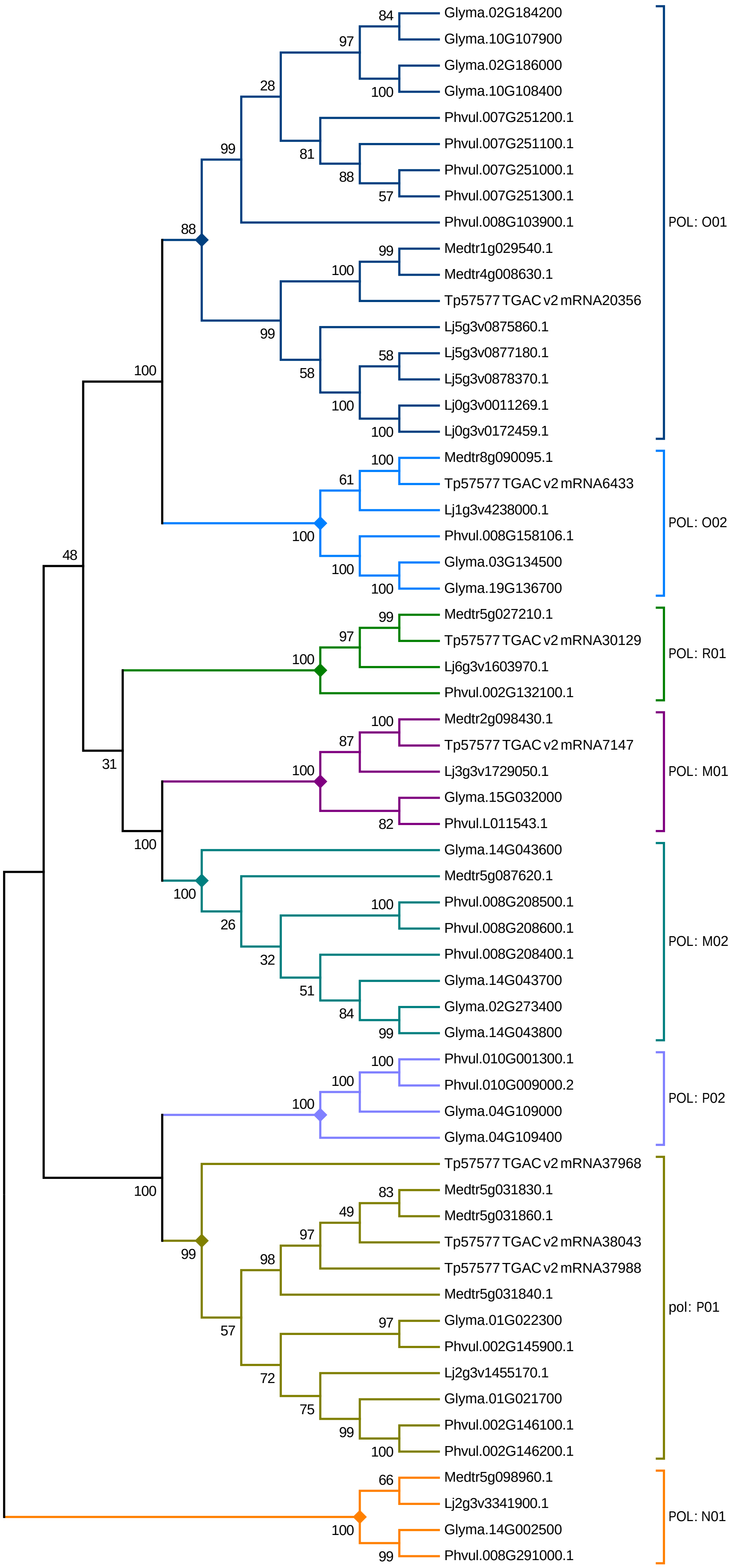

Fig. S11. Putative ortholog loci (POL) in Groups M, N, O, P and R UGTs of Mt, Gm, Pv, Lj and Tp.

The optimal tree with the sum of branch length = 11.60671512 is shown.

- The analysis involved 60 amino acid sequences.
- All ambiguous positions were removed for each sequence pair.
- There were a total of 676 positions in the final dataset.
- Bootstrap percentages (1000 replicates) are shown next to the branches.

Ortholog groupings are shown in different color.

A. Venn diagram of UGT POLs distribution in five legumes

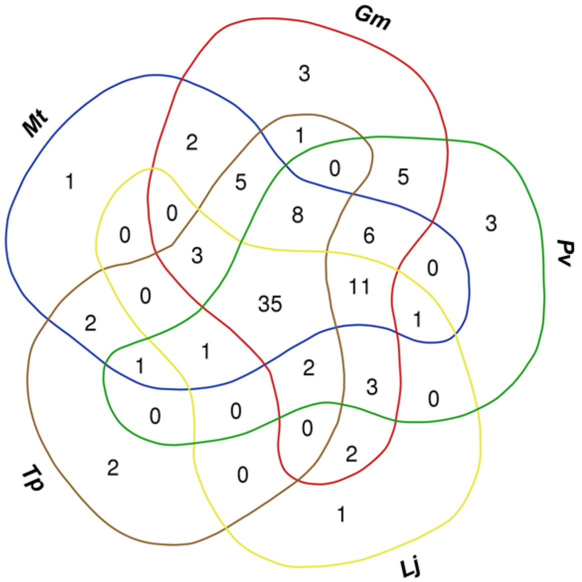

B. Distribution of UGT POLs in five legumes legumes

Species	No. of POLs	POL Ids
Mt, *Gm*, *Pv*, *Lj*, *Tp*	35	A02, A03, A06, A07, B02, B03, D01, D02, D03, D06, D07, D11, D12, E06, E07, E12, E13, E14, E17, E18, G02, G03, O02, H02, I01, J02, L01, L02, L03, L06, L07, L09, O01, M01, P01,
Mt, *Gm*, *Pv*, *Lj*	11	A05, D05, D08, D09, E03, E15, F01, G05, I02, L08, N01
Mt, *Gm*, *Pv*, *Tp*	8	A01, A10, D04, D14, E01, E10, H03, L04
Mt, *Gm*, *Lj*, *Tp*	3	A04, D13, E09
Mt, *Pv*, *Lj*, *Tp*	1	R01
Gm, *Pv*, *Lj*, *Tp*	2	D15, F03
Mt, *Gm*, *Pv*	6	E02, E05, E08, E11, J04, M02
Mt, *Gm*, *Tp*	5	B01, D10 E16, G01, J01
Mt, *Pv*, *Lj*	1	A09
Mt, *Pv*, *Tp*	1	H01
Gm, *Pv*, *Lj*	3	D16, D18, E19
Mt, *Gm*	2	A08, J03
Mt, *Tp*	2	E04, G04
Gm, *Pv*	5	D17, F02, I03, I04, P02
Gm, *Lj*	2	A11, E20
Gm, *Tp*	1	E21
Mt	1	L05
Gm	3	D15, E22, J05
Pv	3	A12, C01, L10
Lj	1	D19
Tp	2	D20, E23

Fig. S12. Distribution of putative ortholog loci (POL) of UGTs in different legumes. A. Venn diagram representation of UGT POLs distribution in legumes. B. Distribution of UGT POLs among legumes. A and B, Mt, Gm, Pv, Lj and Tp implies *Medicago truncatula, Glycine max*, *Phaseolus vulgaris*, *Lotus japonicus*, and *Trifolium pratense* respectively.

Chromosomal distribution of UGTs in *Medicago truncatula* genome

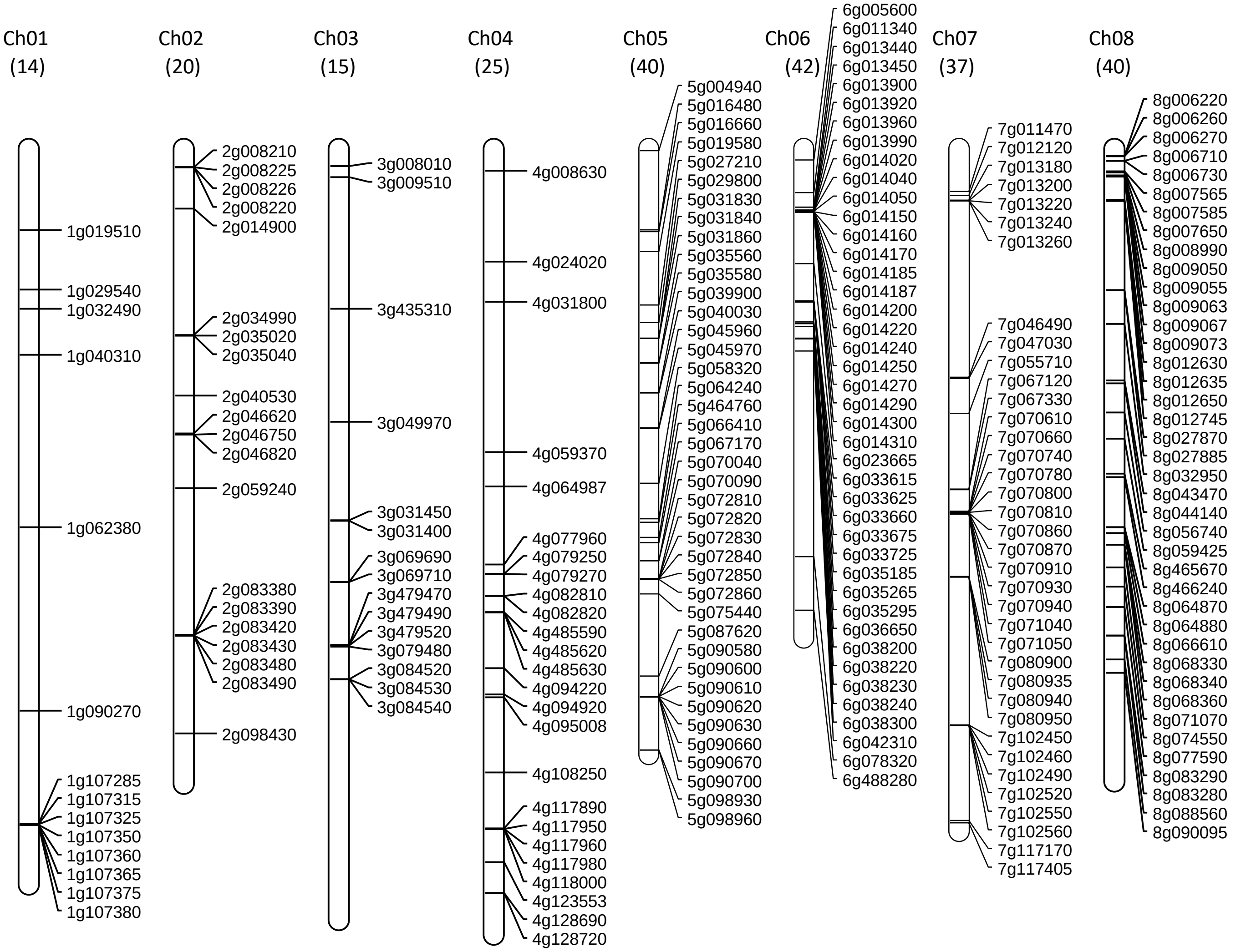

Fig. S13. Genomic distribution of UGTs in *Medicago truncatula* chromosomes. The pseudo-chromosomes (empty vertical bars) numbers (Ch01–Ch08) are mentioned at the top of each vertical bar. Chromosome size is indicated by their relative lengths. Numbers within brackets imply the total number of UGTs identified in the respective chromosome. The physical location of each UGTs is connected by black lines onto the vertical bars. Genes are represented in the figure without their starting identifier (Medtr). Of the 243 MtUGTs, 233 are mapped onto chromosomes and 10 are in scaffolds.

Chromosomal distribution of UGTs in *Glycine max* genome

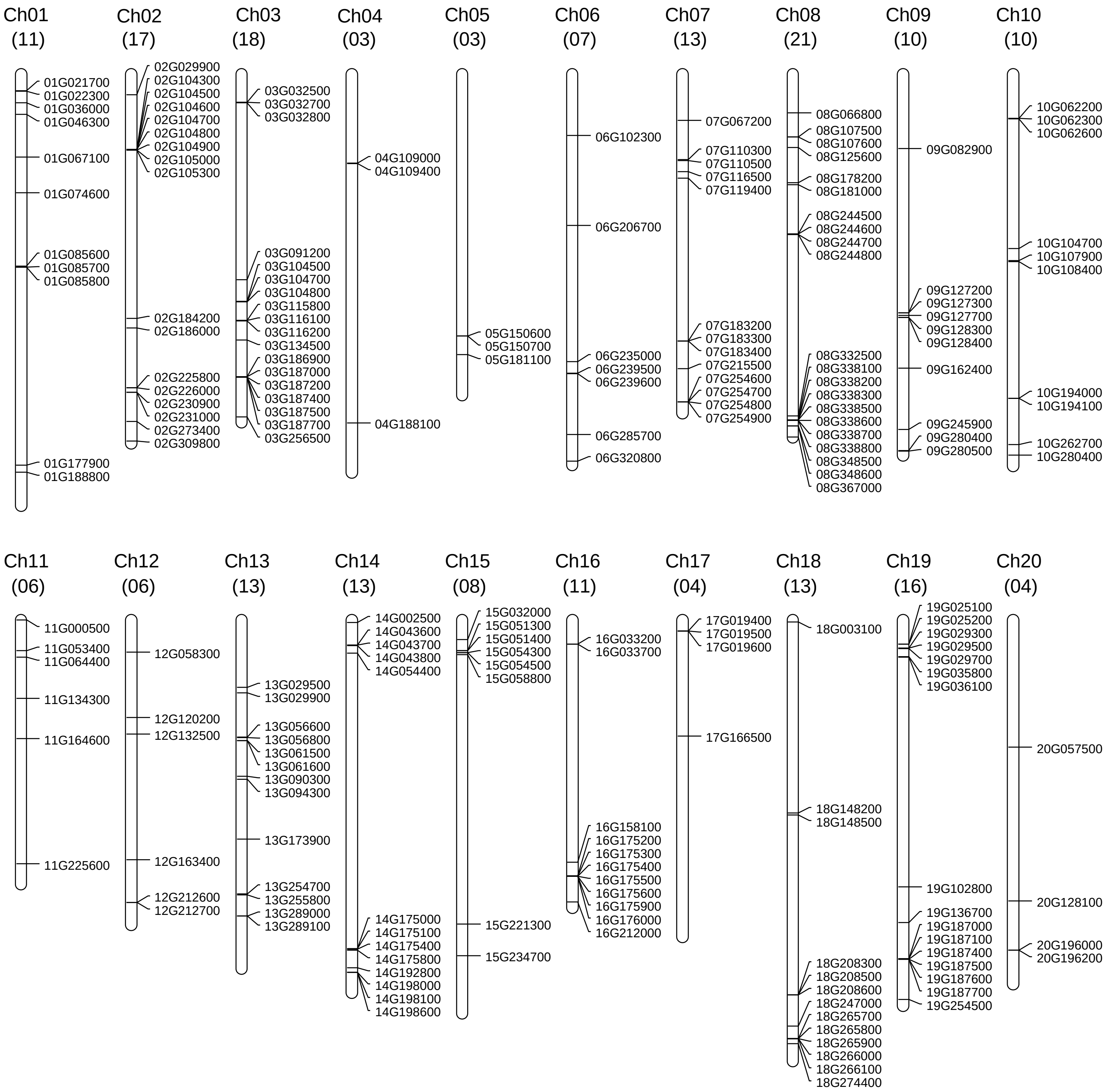

Fig. S14. Genomic distribution of UGTs in *Glycine max* chromosomes. The pseudo-chromosomes (empty vertical bars) numbers (Ch01–Ch20) are mentioned at the top of each vertical bar. Chromosome size is indicated by their relative lengths. Numbers within brackets imply the total number of UGTs identified in the respective chromosome. The physical location of each UGTs is connected by black lines onto the vertical bars. Genes are represented in the figure without their starting identifier (Glyma). Of the 208 GmUGTs, 207 are mapped onto chromosomes and one in scaffold.
