# Supplementary material for: Reconstruction of the Evolutionary Histories of UGT Gene Superfamily in Legumes Clarifies the Functional Divergence of Duplicates in Specialized Metabolism
Source: Int J Mol Sci. 2020 Mar 8;21(5):1855. doi: 10.3390/ijms21051855 (PMC7084467; doi:10.3390/ijms21051855)
Supplement: Supplementary file 1 [file ijms-21-01855-s001.zip › ijms-713495_supllementary files/03. 20200307_Text S1.pdf]

Supplementary Text 1. List of UGTs used to create Figure 1

Notes: Full-length protein sequences are used for the construction of Figure 1**.** See Table S6 for POL assignment and species information

No.	POL No.	UGT Ids		No.	POL No.	UGT Ids
1	A01	Medtr1g090270.1; Glyma.10G194000		50	E13	Medtr0078s0190.1; Glyma.08G338100
2	A02	Medtr2g008220.1; Glyma.08G181000		51	E14	Medtr7g117170.1; Glyma.03G256500
3	A03	Medtr2g008226.1; Glyma.15G051400		52	E15	Medtr8g006260.1; Glyma.07G110300
4	A04	Medtr2g014900.1; Glyma.08G332500		53	E16	Medtr8g006270.1; Glyma.03G116100
5	A05	Medtr2g083380.1; Glyma.13G254700		54	E17	Medtr0184s0030.1; Glyma.08G367000
6	A06	Medtr2g083420.1; Glyma.12G212600		55	E18	Medtr8g074550.1; Glyma.12G163400
7	A07	Medtr4g079270.1; Glyma.06G285700		56	E19	Glyma.03G032800; Lj0g3v0128059.1
8	A08	Medtr8g007565.1; Glyma.07G067200		57	E20	Glyma.05G181100; Lj5g3v0780820.1
9	A09	Medtr7g067330.1; Phvul.010G037600.1		58	E21	Glyma.09G128300; Ca_21871
10	A10	Medtr8g068340.1; Glyma.18G148200		59	E22	Glyma.09G128400; C.cajan_08626
11	A11	Glyma.06G235000; Lj3g3v0428930.1		60	E23	Tp57577 TGAC v2 mRNA40423; C.cajan_28637
12	A12	Phvul.011G136400.1; Vradi0377s00040.1		61	F01	Medtr4g128690.1; Glyma.13G094300
13	B01	Medtr2g059240.1; Glyma.06G206700		62	F02	Glyma.07G183400; Phvul.002G214500.1
14	B02	Medtr0009s0240.1; Glyma.01G067100		63	F03	Glyma.13G255800; Lj6g3v2192300.1
15	B03	Medtr8g466240.1; Glyma.14G054400		64	G01	Medtr2g008210.1; Glyma.15G051300
16	C01	Phvul.008G151900.1; Vradi04g04260		65	G02	Medtr6g014050.1; Glyma.19G035800
17	D01	Medtr1g040310.1; Glyma.15G221300		66	G03	Medtr3g079480.1; Glyma.13G029500
18	D02	Medtr5g045970.1; Glyma.10G062300		67	G04	Medtr4g094220.1; Aradu.598J8.1
19	D03	Medtr4g117950.1; Glyma.07G254700		68	G05	Medtr5g464760.1; Glyma.14G175100
20	D04	Medtr4g024020.1; Glyma.03G187500		69	H01	Medtr3g069690.1; Phvul.001G238800.1
21	D05	Medtr4g031800.1; Glyma.16G033700		70	H02	Medtr3g069710.1; Glyma.11G225600
22	D06	Medtr7g070940.1; Glyma.09G280400		71	H03	Medtr4g064987.1; Glyma.11G134300
23	D07	Medtr8g066610.1; Glyma.02G105000		72	I01	Medtr7g013180.1; Glyma.18G265700
24	D08	Medtr4g485630.1; Glyma.02G104600		73	I02	Medtr7g013240.1; Glyma.08G244800
25	D09	Medtr5g016660.1; Glyma.11G053400		74	I03	Glyma.01G085600; Aradu.177E7.1
26	D10	Medtr5g040030.1; Glyma.01G046300		75	I04	Glyma.19G029300; Aradu.JG94E.1
27	D11	Medtr5g045960.1; Glyma.19G187700		76	J01	Medtr3g031400.1; Glyma.11G164600
28	D12	Medtr7g117405.1; Glyma.03G186900		77	J02	Medtr5g072830.1; Glyma.14G198000
29	D13	Medtr7g102550.1; Glyma.19G187500		78	J03	Medtr5g075440.1; Glyma.02G230900
30	D14	Medtr7g102560.1; Glyma.03G187700		79	J04	Medtr5g072860.1; Glyma.02G231000
31	D15	Glyma.01G188800; Lup001002.1		80	J05	Glyma.04G188100; Ca_08995
32	D16	Glyma.02G104300; Lj4g3v0620410.1		81	L01	Medtr1g032490.1; Glyma.20G128100
33	D17	Glyma.10G062200; Lj5g3v0681080.1		82	L02	Medtr1g107380.1; Glyma.10G262700
34	D18	Glyma.08G348500; Phvul.008G130100.2		83	L03	Medtr8g083290.1; Glyma.05G150600
35	D19	Glyma.11G000500; Lj1g3v0116690.1		84	L04	Medtr4g094920.1; Glyma.17G166500
36	D20	Lj5g3v0681090.1; Aradu.GH7AM.1		85	L05	Medtr5g004940.1; C.cajan_29587
37	D21	Tp57577 TGAC v2 mRNA6677; GenBank: GAU45636.1_T. subterraneum		86	L06	Medtr5g035560.1; Glyma.01G036000
38	E01	Medtr1g019510.1; Glyma.03G104500		87	L07	Medtr6g038300.1; Glyma.16G158100
39	E02	Medtr2g046750.1; Glyma.09G082900		88	L08	Medtr7g080935.1; Glyma.18G247000
40	E03	Medtr3g009510.1; Glyma.06G320800		89	L09	Medtr8g088560.1; Glyma.08G125600
41	E04	Medtr4g082820.1; Tp57577 TGAC v2 mRNA41332		90	L10	Phvul.011G184600.1; Vradi0371s00010.1
42	E05	Medtr5g019580.2; Glyma.01G177900		91	M01	Medtr2g098430.1; Glyma.15G032000
43	E06	Medtr5g070040.1; Glyma.14G192800		92	M02	Medtr5g087620.1; Glyma.14G043700
44	E07	Medtr5g090660.1; Glyma.02G309800		93	N01	Medtr5g098960.1; Glyma.14G002500
45	E08	Medtr6g005600.1; Glyma.07G110500		94	O01	Medtr1g029540.1; Glyma.02G184200
46	E09	Medtr8g465670.1; Glyma.03G116200		95	O02	Medtr8g090095.1; Glyma.03G134500
47	E10	Medtr6g033615.1; Glyma.16G175300		96	P01	Medtr5g031830.1; Glyma.01G022300
48	E11	Medtr6g078320.1; Glyma.16G212000		97	P02	Glyma.04G109000; Phvul.010G001300.1
49	E12	Medtr7g046490.1; Glyma.01G074600		98	R01	Medtr5g027210.1; Phvul.002G132100.1
